# A human mesenchymal spheroid prototype to replace moderate severity animal procedures in leukaemia drug testing

**DOI:** 10.12688/f1000research.123084.1

**Published:** 2022-11-09

**Authors:** Aaron Wilson, Sean Hockney, Jessica Parker, Sharon Angel, Helen Blair, Deepali Pal

**Affiliations:** 1 Wolfson Childhood Cancer Research Centre, Translational and Clinical Research Institute, Newcastle University, UK, Newcastle upon Tyne, NE1 7RU, UK; 2 Applied Sciences, Northumbria University, Newcastle upon Tyne, NE1 8ST, UK

**Keywords:** Animal replacement, 3D models, Preclinical models, cancer research, leukaemia

## Abstract

Patient derived xenograft (PDX) models are regarded as gold standard preclinical models in leukaemia research, especially in testing new drug combinations where typically 45-50 mice are used per assay. 9000 animal experiments are performed annually in the UK in leukaemia research with these expensive procedures being classed as moderate severity, meaning they cause significant pain, suffering and visible distress to animal’s state. Furthermore, not all clinical leukaemia samples engraft and when they do data turnaround time can be between 6-12 months. Heavy dependence on animal models is because clinical leukaemia samples do not proliferate *in vitro.* Alternative cell line models though popular for drug testing are not biomimetic – they are not dependent on the microenvironment for survival, growth and treatment response and being derived from relapse samples they do not capture the molecular complexity observed at disease presentation. Here we have developed an *in vitro* platform to rapidly establish co-cultures of patient-derived leukaemia cells with 3D bone marrow mesenchyme spheroids, BM-MSC-spheroids.  We optimise protocols for developing MSC-spheroid leukaemia co-culture using clinical samples and deliver drug response data within a week. Using three patient samples representing distinct cytogenetics we show that patient-derived-leukaemia cells show enhanced proliferation when co-cultured with MSC-spheroids. In addition, MSC-spheroids provided improved protection against treatment. This makes our spheroids suitable to model treatment resistance – a major hurdle in current day cancer management

Given this 3Rs approach is 12 months faster (in delivering clinical data), is a human cell-based biomimetic model and uses 45-50 fewer animals/drug-response assay the anticipated target end-users would include academia and pharmaceutical industry. This animal replacement prototype would facilitate clinically translatable research to be performed with greater ethical, social and financial sustainability.

Research highlights **Scientific benefit**

A 3D spheroid-based approach with improved clinical relevance for *ex vivo* co-culture of clinical leukaemia samples for further investigation into cancer biology such as blast-niche interactions, blast proliferation and treatment resistance.

**3Rs benefit**

To replace moderate severity animal (mice) procedures in leukaemia research and drug testing.

**Practical benefit**

3Rs approach that yields drug response data quickly and is more ethically, socially and financially sustainable than its *in vivo* counterparts.

**Current applications**

Exploration of leukaemia biology such as blasts proliferation, blast-niche interactions, niche-impacted treatment resistance and obtain drug response data.

**Potential applications**

Adapt the approach to include other haematological cancers as well as bone cancers.

## Introduction

Leukaemia management is hindered by two chief obstacles: 1. Treatment still involves toxic chemotherapy drugs ^1^^,^^2^ 2. 15-20% of patients go into relapse at which point the disease can be treatment resistant. ^3^ The nature of relapse disease highlights the need for safer and improved treatments such as targeted therapy. While many novel therapeutics have been identified, high drug attrition rates have been a major hindrance against anti-cancer drug development. 95% of bench side drugs that make it to phase 1 clinical trial never reach patients. ^4^^–^^6^ Three key areas ^5^ have been identified that must be addressed in order to reduce attrition rates: 1. Increase in scientific collaboration 2. Preclinical studies and clinical trials must be tumour biology driven for the drug to be more therapeutically effective. 3. Lack of robust and translatable preclinical cancer models that could be used to screen out many unsuccessful therapeutics before going to trial. ^5^

The gold standard preclinical model in leukaemia research is a patient derived xenograft model. ^7^^–^^9^ Recent Home Office annual returns show that 195,407 mice are used annually in cancer research. Indeed, cancer research remains the most common applied sciences area to use animal procedures. Given leukaemia research is heavily dependent on mouse models combining these metrics with financial data from major cancer funders in the UK suggest that 5% of these mice or at least 9000 mice are used in leukaemia research every year. Although leukaemia is a very aggressive disease, leukaemia cells do not maintain viability outside the body. ^9^ This has led to the development of patient-derived xenograft (PDX) models where leukaemia cells are engrafted into the BM of immunocompromised mice. PDX models are currently the most clinically relevant models in blood cancer preclinical studies. Mouse PDX models (which are regarded as moderate severity procedures) are used to amplify patient-derived leukaemia cells, to study leukaemia biology and perform translational studies. ^8^^,^^10^ Patient-derived leukaemia cells can take anywhere between 2-12 months to engraft in mice before any preclinical drug testing can be carried out, hence these mice experiments are very time consuming. In addition, most of these procedures are considered to be moderate severity and often involve mice developing significant adverse effects such as weight loss, fur ruffling, back hunching as well as increased white cell counts.

The pipeline for preclinical testing using mouse PDX models are as documented in literature is: 1. Toxicity analysis to determine no observed adverse effect limit (NOAEL)/maximum tolerated dose (MTD): 15-20 mice per drug. ^11^ 2. Pharmacokinetic studies to determine drug maximum plasma/tissue levels and kinetics of drug clearance:15-30 mice per drug. ^12^ 3. Drug efficacy studies and pharmacodynamics assays on tumour cell response: 5-10 mice needed per drug treatment group. ^13^ Per drug group a total of 40-50 animals are needed for single drug assays and twice as many for drug combination assays. ^11^^–^^13^ In addition, *in vitro* cancer models preceding *in vivo* validation experiments rely on cell lines that show limited *in vivo* predictability. Cell lines retain cytogenic characteristics of acute lymphoblastic leukaemia (ALL), but being derived from patients with relapsed disease these samples do not represent the molecular complexity of disease at presentation. ^14^^,^^15^ Cell lines have also artificially adapted to grow in suspension culture which eliminates a key feature of leukaemia biology: microenvironment and its role in treatment resistance. Furthermore, these models are restricted by limited *in vivo* predictability and often lead to *in vitro* to *in vivo* drug attrition rates. This in turn results in unnecessary animal experiments which could have been avoided for these failed drug candidates, i.e., false positive data from cell line models.

ALL is a disease primarily of the bone marrow (BM) microenvironment (or leukemic niche), a compartment within the bone composed of cell types including mesenchymal stroma cells (MSC), osteoblasts, osteoclasts, endothelia, immune cells and supporting perivascular cells. Difficulty in growing and maintaining patient derived samples once they removed from the patient alludes to the vital role that the niche plays in cancer biology. Study into blast-niche interaction has unveiled some of the supportive roles the niche plays in disease, including cell-cell signalling, growth factors, cell adhesion and evidence has shown the niche providing an element of chemoprotection. ^16^^,^^17^ The niche particularly mesenchyme-blast interaction gives rise to two lymphoblast subpopulations, the first being a slow cycling, dormant population and the second being actively cycling disease propagating blasts. ^14^^,^^18^ Many treatments are effective in reducing the populations of active cycling blasts; however, it is reported that the dormant populations can survive treatment, seeking refuge within the niche. These dormant cells can later shift into the cycling state post treatment, leading to relapse disease. ^15^^,^^18^

Targeting of niche-blast interactions opens a door into a variety of potential therapeutic approaches. An example would be CXCL12, a chemokine secreted by stroma cells and upregulated in disease, it was discovered that CXCL12 induces a non-cycling state within LSCs, reducing the effectiveness of tyrosine kinase inhibitors. Mesenchymal cell CXCL12 knockout reversed this change and caused a phenotypic switch into disease propagating cycling blasts, temporarily increasing disease load but allowing once chemo resistant blasts to be treated. ^19^ In preceding studies, we have developed *ex vivo* 2D co-culture platforms, ^14^^,^^15^ the purpose of which has been to 1. conduct preclinical drug screening using patient-derived leukaemia cells thereby reducing dependence on cell lines and consequently minimising *in vitro* to *in vivo* drug attrition rates and 2. Explore the impact of human BM cells in driving key elements of cancer biology such as leukaemia proliferation, dormancy and treatment resistance. ^14^ Importantly, to build proof of confidence in application we show that such human relevant *ex vivo* platforms have 1. the potential to detect therapeutically exploitable targets that disrupt leukaemia-BM cell interactions conferring treatment resistance to the cancer cells. For example, using our *ex vivo* approach we reveal that CDH2 drives niche-mediated treatment resistance in leukaemia and that this interaction can be targeted via a drug ADH-1. In addition, we validate that CDH2 is indeed significantly upregulated in human leukaemia bone marrow thereby corroborating the strength of our *ex vivo* model in predicting human/clinical data. Such models also have greater experimental accessibility than *in vivo* procedures and thus mitigate reliance on mouse experiments to perform such mechanistic studies 2. We use our *ex vivo* approach to conduct ADH-1 drug testing on 15 different patient-derived clinical samples (i.e., biological replicates. We used previously cryopreserved PDX clinical samples generated historically in older projects) and drug combination testing on 3 patient-derived clinical samples. These *ex vivo* experiments informed our *in vivo* validation experiments so these could be streamlined to achieve optimal animal replacement. Although such 2D studies aim to successfully capture interaction of patient-derived leukaemia cells with cell components of the human BM niche/leukaemia microenvironment, 2D models do not portray functional biomimicry and consequently improved clinical relevance displayed by 3D models.

Recent advances in medicine have utilised 3D models as miniature *in vitro* representations of specific organs, they are produced in 3D and retain a realistic microanatomy, allowing a representational human model. ^20^ 3D models recapitulate their respective organ function and have been proven to be useful models of human disease, particularly in cancer research. ^21^ 2D models cannot recapitulate the leukemic niche or the complex and varied interactions that influence leukemic cell growth. ^22^ Current medicine is making use of organ-on-a-chip systems which have led to advances in liver and lung drug screening, providing more accurate toxicological reports than 2D systems. ^20^ The complex and dynamic nature of ALL and the bone marrow mesenchymal microenvironment makes it an ideal candidate for a more representative drug screening platform, this paper aims to do this by developing mesenchymal spheroids, which will support the culture of patient derived leukaemia samples allowing patient-specific therapeutic investigation. ^23^

## Methods

### Materials

**Ethical approval**

Patient-derived leukaemia blasts were obtained from the Newcastle Biobank (REC reference number 07/H0906/109+5). All samples were obtained following written informed consent. Animal data shown here reflect unpublished data that were existing from past experiments. PDX samples used in this project are from our cryopreserved PDX bank where the samples were generated in previous/earlier projects. All animal studies were carried out in accordance with UK Animals (Scientific Procedures) Act, 1986 under project licence P74687DB5 following approval of Newcastle University animal ethical review body (AWERB).

### 2D Niche-Blast co-culture

Patient derived ALL samples were cultured on 2D monolayers of MSC cells. MSC were seeded on Matrigel coated 48 well plates at 2×10 ^4^ cells/0.5 mL/2 cm ^2^ in their respective media (MSC media, Table 1). After 24 hours, the media was aspirated and the cells rinsed with 1 mL SFEM, Leukaemia blasts were then plated at a density of 0.25×10 ^5^ cells/mL per well suspended in SFEMII.

**Table 1.** Materials used.

Material	Manufacturer	Catalogue Identifier
**General**		
Trypsin-EDTA Solution 10X	Sigma-Aldrich, Dorset, UK	59418C
Phosphate-Buffered Saline	Thermo Fisher Scientific, Hertfordshire, UK	10010023
Gentle Cell Dissociation Reagent	Stem Cell Technologies, Stem Cell, UK	**100-0485**
Heat Inactivated Foetal Bovine Serum	Thermo Fisher Scientific, Hertfordshire, UK	10500064
Matrigel HESC-Qualified Matrix	Corning, Kennebunk, USA	CLS354277
Low-glucose Dulbecco's Modified Eagle's Medium	Sigma-Aldrich, Dorset, UK	D5546
**Cells**		
Primary Mesenchymal Stem Cells	Obtained from patients undergoing total hip replacement in view of osteoarthritis	
PDX sample L707	Obtained from patient with E2A-HLF translocation	
PDX sample L49120	Obtained from patient with BCR-ABL translocation	
PDX sample MS40	Obtained from patient with MLL rearrangement	
**MSC culture media**		
Low-glucose Dulbecco's Modified Eagle's Medium	Sigma-Aldrich, Dorset, UK	D5546
Heat Inactivated Foetal Bovine Serum	Thermo Fisher Scientific, Hertfordshire, UK	10500064
Penicillin/Streptomycin	Sigma-Aldrich, Dorset, UK	P4333
Basic FGF	Sigma-Aldrich, Dorset, UK	PHG0024
L-Glutamine	Sigma-Aldrich, Dorset, UK	59202C
Matrigel HESC-Qualified Matrix	Corning, Kennebunk, USA	CLS354277
**Leukaemia Blast Co-Culture**		
SFEM, Serum-Free Medium for Culture and Expansion	Stem Cell Technologies, Stem Cell, UK	09650
**Organoid formation**		
AggreWellM 400 24-well Plate	Stem Cell Technologies, Cambridge, UK	34411
AggreWell Rinsing Solution	Stem Cell Technologies, Cambridge, UK	**07010**
Costar Ultra-low Attachment 6 Well Plate	Corning, Kennebunk, USA	3471
**Real-Time PCR Analysis**		
QIAShredder	Qiagen, Manchester, UK	79656
Beta-mercaptoethanol	Sigma-Aldrich, Dorset, UK	M6250
RNEasy Mini Kit	Qiagen, Manchester, UK	74104
RNase-Free DNase Set	Qiagen, Manchester, UK	79254
**Primer Sequences**		
CDH2 forward primer, Sigma-Aldrich, Dorset, UK	GGTGGAGGAGAAGAAGACCAG	OLIGO
CDH2 reverse primer, Sigma-Aldrich, Dorset, UK	GGCATCAGGCTCCACAGT	OLIGO
CD90 forward primer, Sigma-Aldrich, Dorset, UK	CACACATACCGCTCCCGAAC C	OLIGO
CD90 reverse primer, Sigma-Aldrich, Dorset, UK	GCTGATGCCCTCACACTT	OLIGO
NES forward primer, Sigma-Aldrich, Dorset, UK	AGAGGGGAATTCCTGGAG	OLIGO
NES reverse primer, Sigma-Aldrich, Dorset, UK	CTGAGGACCAGGACTCTCTA	OLIGO
GAPDH forward primer, Sigma-Aldrich, Dorset, UK	GAAGGTGAAGGTCGGAGTC	OLIGO
GAPDH reverse primer, Sigma-Aldrich, Dorset, UK	GAAGATGGTGATGGGATTTC	OLIGO

### 3D culture of BM-MSC

MSC cells were transferred to a 6-well low adhesion plate for suspension culture and cultured in 2 mL MSC media ( Table 1) 3D for 48 hours, after which, MSC spheres were seen to form. Spheroids obtained thus were irregular in shape and therefore optimised experiments using Aggrewell400 plates were used to create uniform MSC spheres. Aggrewell plates were prepared by rinsing each well with 0.5 mL of Aggrewell rinsing solution, spinning at 1500 g for 5 minutes and aspirating. MSC cells were seeded at varying concentrations depending on desired sphere size. Cells were split using 1x trypsin solution and seeded in single cells on Aggrewell plates at desired concentration in MSC media. Aggrewell plates were spun at 100g for 3 minutes to ensure all cells reached the bottom of the microwells. After 48 hours, MSC spheroids were ready for harvest and transferred manually for culture onto low adhesion plates. Using a stereomicroscope fitted within a Class II biological safety cabinet the spheroids were transferred using a Gilson p1000 pipette tip with the end cut off using sterile scissors.

### 3D BM-Blast co-culture

Leukaemia cells from PDX samples were co-cultured with the MSC spheres in SFEMII at 2×10 ^5^ cells/mL in 5 ml of media in low adhesion 48 well plates over a 5 day period. On day of harvest, the cells and spheroids were harvested into a 30 ml conical flask. This was centrifuged at 1500 RPM for 5 minutes. After aspirating the supernatant, 0.5 ml of X1 Trypsin EDTA was added and cultures incubated at 37°C, 5% CO _2_ for 2 minutes. Cells were mechanically dissociated into single cells by resuspending with a pipette. To the cells 4ml of FBS was added and this was centrifuged at 1500 RPM for 5 minutes. Supernatant was aspirated and cells were resuspended in 1ml of fresh SFEM media. During cell count leukaemia cells were distinguished from MSC based on their morphology and size difference, the MSC are 3-4 times the size of leukaemia cells.

### Dexamethasone 3D drug assay

Investigation into Dexamethasone mediated treatment resistance was carried out through cell fate tracing. 10 million blasts were span at 500 g and resuspended in 10 mL of 5 uM CellTrace violet, incubated for 20 minutes at 37 degrees Celsius, 1 mL FBS was added and cells were span once more at 500 g for 5 minutes and resuspended in fresh SFEMII. Cells were then plated on 3D MSC spheres in 48 well plates as described above. 5 nM Dexamethasone (stock dissolved in ethanol to generate 10 mM Dexamethasone concentration, working concentration of dexamethasone was made by performing serial dilutions using SFEM media) was added to desired wells and blasts were cultured for 5 days. Experiments include 3 biological replicates with each replicate including 3 experimental replicates.

### Extraction of mRNA

RNA was purified from cell pellets (viable cells centrifuged at 5000 g × 5 minutes followed by aspiration of supernatant media) using the RNeasy mini kit. Cells were resuspended in buffer RLT plus (containing 10 μl beta-mercaptoethanol/10 ml RLT) at either <5×10 ^6^ cells/350 μl buffer RLT plus or 1×10 ^7^ cells/600 μl buffer RLT plus. 1 volume of 70% ethanol was added to the RNA lysate and 700 μl of the cell suspension was transferred to a RNeasy mini spin column placed in a 2 ml collection tube, this was centrifuged at maximum speed for 30 seconds and the flow through discarded. The RNase-free DNase kit was used to remove contaminating DNA. 10 μl DNase I stock solution was added to 70 μl buffer RDD and mixed by gently inverting the tube, this solution was added directly to the column membrane and the sample incubated at room temperature for 15 minutes. 350 μl buffer RW1 was added and centrifuged at maximum speed for 30 seconds, the flow through was discarded. Next, 500 μl buffer RPE was added to the spin column, centrifuged at maximum speed for 30 seconds, and flowthrough discarded. Another 500 μl buffer RPE was added to the spin column, centrifuged at maximum speed for 2 minutes and the flow through discarded. The RNeasy spin column was transferred to a new 2 ml collection tube and centrifuged at maximum speed for 1 minute with the column lid open to dry the membrane. 30 μl RNase free water was then added to elute the sample, centrifuged for 1 minute at maximum speed and the flow through was collected. The RNA yield and quality was checked using the nanodrop ND-1000 spectrophotometer.

### cDNA synthesis

RevertAidTMH Minus First Strand cDNA Synthesis Kit was used to synthesise cDNA from the RNA isolated. 500 ng RNA was collected and added to RNase/DEPC free water to a final volume of 11 μl. 1 μl (dN)6 (200 mg/l) random hexamers was added, mixed gently by inverting the vial and briefly centrifuged. Using a GeneAmp PCR system 2700 the sample was incubated at 65°C for 5 minutes, after which the sample was immediately placed on ice, 8 μl of the master mix ( Table 2) was added, the samples were vortexed and briefly centrifuged. The samples were placed back in the PCR machine to incubate at 25°C for 10 minutes, 42°C for 60 minutes and 75°C for 10 minutes to terminate the reaction. The product was either used immediately for RT-PCR or stored at -20°C for short term storage.

**Table 2.** cDNA master mix constituents.

Reagents	Volume (μl)
5x Reaction Buffer	4
RNase Inhibitor	1
10mM dNTP	2
RevertAid H Minus MMLV RT	1
**Total Volume**	**8**

### qRT-PCR

Upon receipt, primers were reconstituted in RNase/DNase free water to a final stock concentration of 100 μM. PCR Mix ( Table 3) was loaded onto PCR plate in triplicates. The plate was sealed and centrifuged for 1 minute at 1000 RPM and placed in an applied Biosystems 7900HT Sequence Detection System. ViiA7TM System was used to run the qRT-PCR plate and cycle threshold (Ct) and melting curves were obtained for analysis.

**Table 3.** qRT-PCR mix constituents.

Reagents	Volume (μl)
SYBR Green	5
10μM Working Primer	0.3
RNase-free water	2.7
cDNA Sample	2
**Total**	**10**

### Costings analysis of *in vivo* versus *in vitro* models

Costings of *in vitro* models were estimated using list prices of media, reagents and other materials associated with developing and using the models for a 2 drug combination dose finding assay. This included 5 biological replicates, 3 experimental repeats with each having 3 technical repeats with a total number of experiments of 45. These costs were divided into the following categories: Production of clinical/patient-derived leukaemia samples; *in vitro* CRISPR/RNAi organoid generation; Dose/IC50 finding for 2 drug combination; and evaluation of drug combination.

*In vivo* costings were calculated using figures obtained from the Comparative Biology Centre, Newcastle University. This included fixed costs including mouse purchase from an NSG in-house colony, housing and IVIS use. Comparative *in vivo* equivalents to the *in vitro* design accounted for: production of PDX engrafted tissue (5 PDX, 6 NSG mice/PDX); *in vivo* CRISPR/RNAi (control and treatment groups, 5 NSG mice each); Dose finding for 2 drug combination (3 mice per sex); and evaluation of the combination (4 arms, 6 mice per arm). All total costings were calculated using Microsoft Excel and figures were created using Microsoft Excel.

### Data analysis

Microscopy images were analysed using the Windows download bundled with Java 8. Version 1.4.3. Real time qPCR data were captured and analysed using the StepOne Plus Real-Time PCR System (ThermoFisher Catalog No: 4376600) and Software package StepOne Software v23.

## Results

### Development and validation of BM-MSC spheres

**Figure 1.**
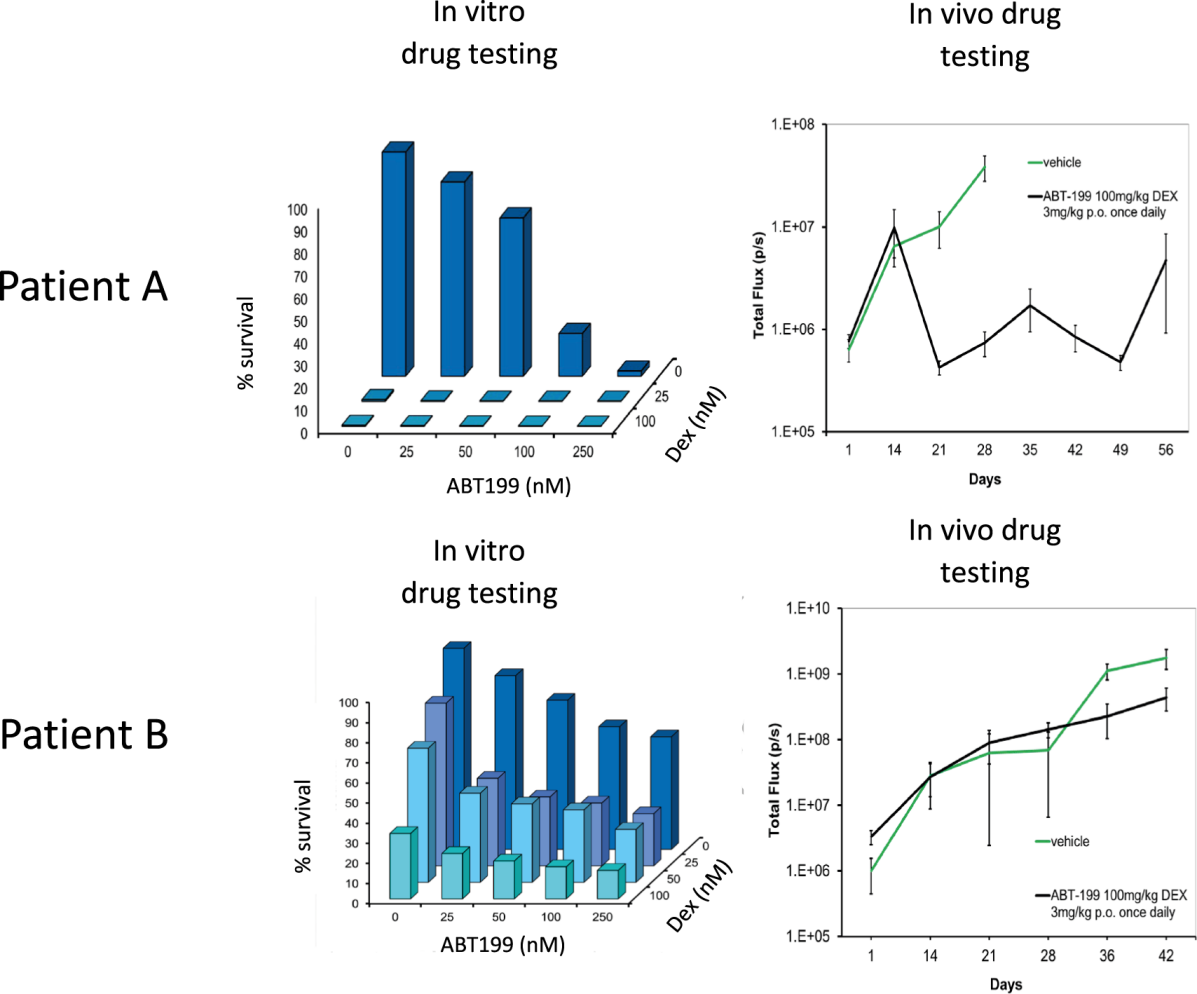
2D *in vitro* platform delivers drug response data with high *in vivo* predictability to minimise *in vitro* to *in vivo* drug attrition rates. Leukaemia cells from patient A is a responder to ABT199 (targets BCL2 in BCL2 positive ALL) and Dexamethasone drug combination *in vitro* and *in vivo.* On the other hand leukaemia cells from patient B do not respond to this drug combination *in vitro* and subsequently poor response is also noted *in vivo* using PDX mice models. Error bars shown refer to standard error (SE) using 4 mice per treatment group. Total flux is a surrogate of blast number *in vivo.* Dexamethasone and ABT-199 are used in the clinics to treat ALL.

**Figure 2.**
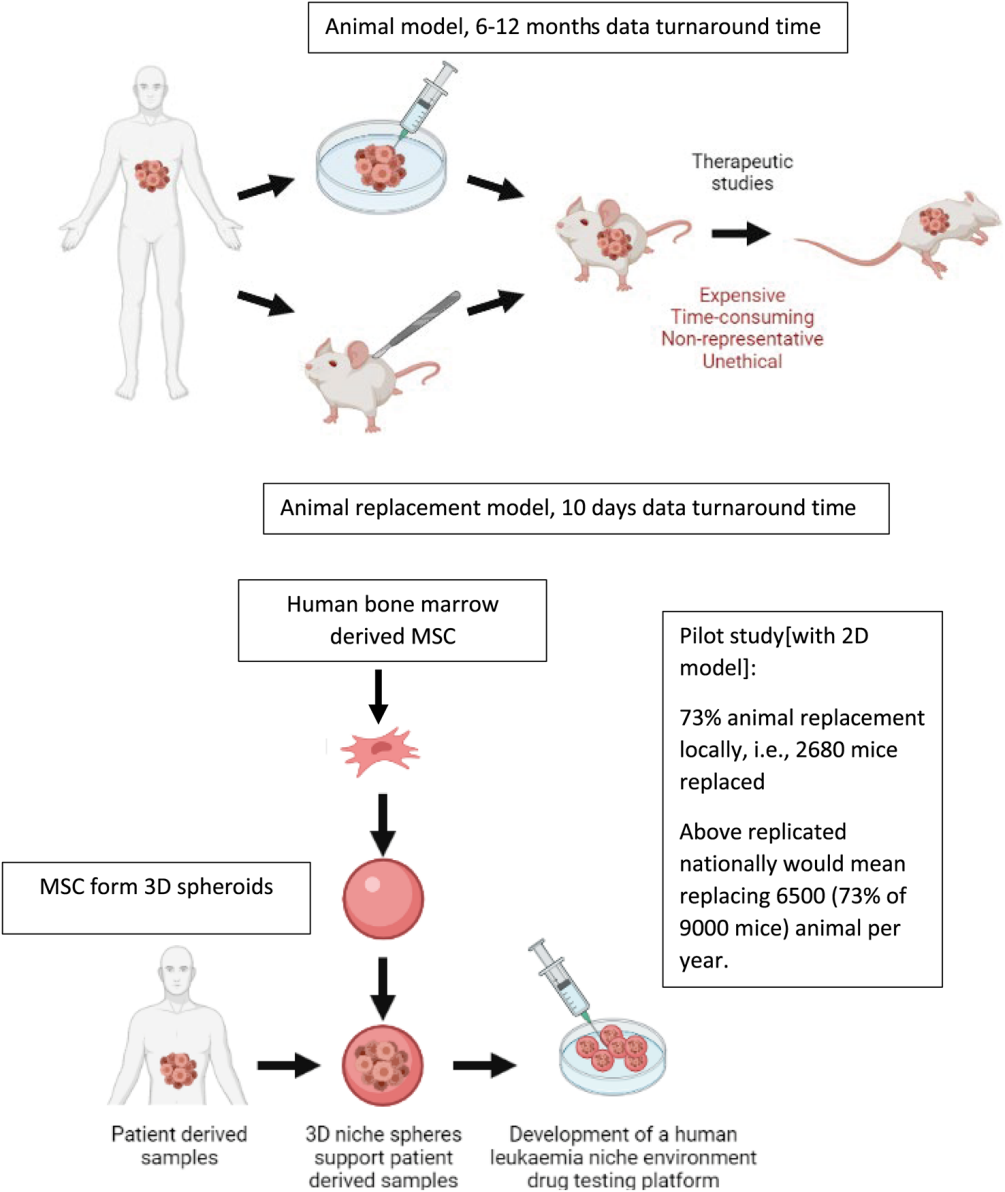
Comparison of existing *in vivo* preclinical leukaemia models with newly developed *ex vivo* animal replacement organoid models. Key benefits of the animal replacement organoid models include species specificity and consequently higher biomimicry.

**Figure 3.**
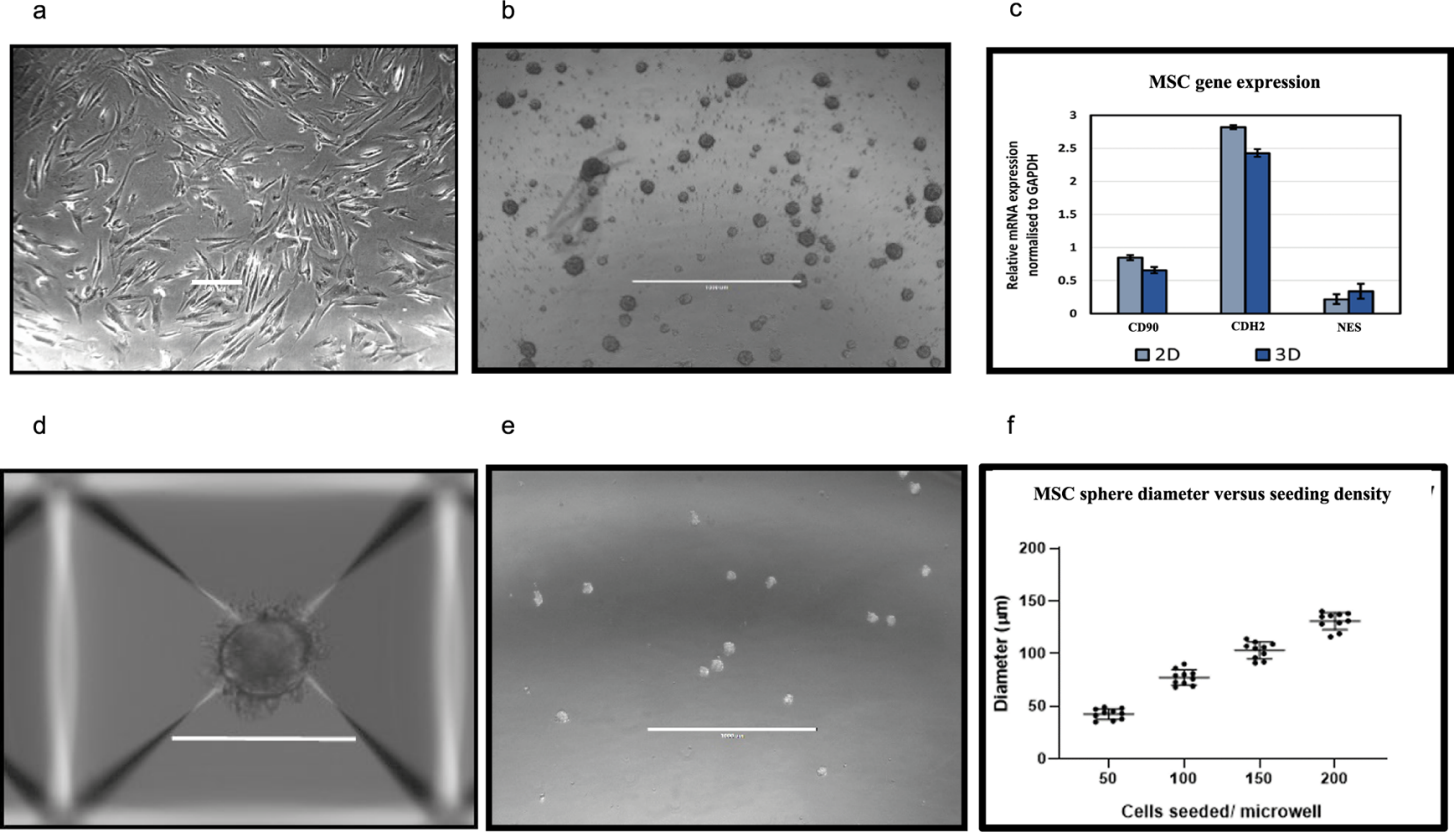
Advancing the 2D *ex vivo* platform to develop 3D spheroid models. Development and validation of MSC spheroid cultures through 3D suspension culture. Aggrewell plates allow for the creation of uniform 3D spheroids from single cell suspension of the desired cell-type. SD from three independent experiments (using MSC from same patient) are plotted as error bars. 2D (a), scale bar = 100 μM and 3D MSC (b), scale bar = 200 μM after 96 hours of growth. (c) mesenchymal marker levels quantified through qPCR and normalised to GAPDH. **(d,e)** MSC spheroids obtained through Aggrewell plate culture. Scale bar = 1000 μM (f) Cell seeding densities directly correlate to size of spheroids obtained. Data captured following 48 hours of seeding.

We first show that BM-MSC/leukaemia 2D co-culture platform generates drug dose response data that is comparable with drug response observed *in vivo* in mouse models ( Figure 1). ^24^ These data where we compare our *in vitro* data with *in vivo* data (unpublished data existing in the lab from earlier studies) show the predictive capacity of our 2D co-culture platform against mouse experiments. Improved *in vivo* predictability is important in *ex vivo* animal replacement models if these platforms are to meaningfully replace mouse experiments. We further perform pilot studies where we show that such *ex vivo* co-culture platforms have the ability to bring about significant replacement of animals for PDX models, which are the current gold standard preclinical leukaemia model ( Figure 2). In order to fully recapitulate the complexity of the leukaemia niche, a 3D Model with improved biomimicry is required. 3D BM-MSC-spheroids were created by subculturing cells onto low adhesion non-tissue culture treated plates ( Figure 3a, b). These spheroids were tested for expression levels of their respective niche markers using QT-PCR. It was found that both niche sphere types expressed mesenchymal markers CD90, CDH2 and Nestin ( Figure 3c) similar to their 2D counterparts.

Aggrewell400 plates are designed for higher throughput and more uniform production of MSC spheres. Each well on these plates house 400 conical microwells, funnelling single cells and encouraging them to conglomerate into spheroids ( Figure 3d). These microwells were utilised to create uniform niche spheres allowing standardisation of the technique when producing assays involving 3D spheres ( Figure 3e). A direct correlation was observed with seeding cell density and size of spheroids with higher seeding densities resulting in generation of more uniformly sized spheroids ( Figure 3f).

### Patient-derived leukaemia cells co-cultured with MSC show superior proliferation and enhanced treatment resistance

**Figure 4.**
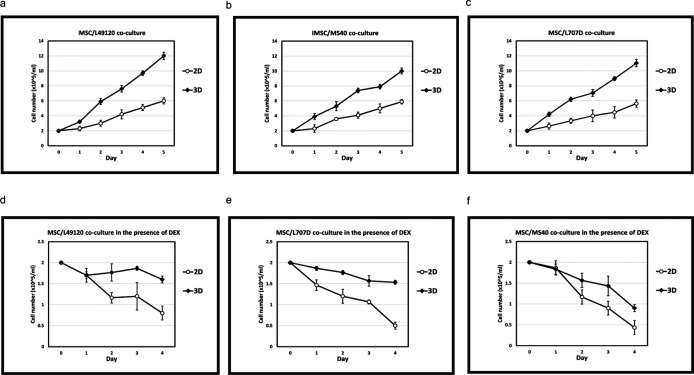
Co-culture of patient derived leukaemia samples on both 2D and 3D MSC +/- treatment pressure. N= 3 Error bars = SD. Increased growth kinetics of L49120 (a,d), MS40 (b,e) and L707D (c,f) across a 5 day period on 3D MSC feeder cells compared to a 2D monoculture. L49120, MS40 and L707D refer to three different patient samples, i.e., 3 biological repeats. Dexamethasone treatment is less effective in a 3D microenvironment (d,e,f).

**Figure 5.**
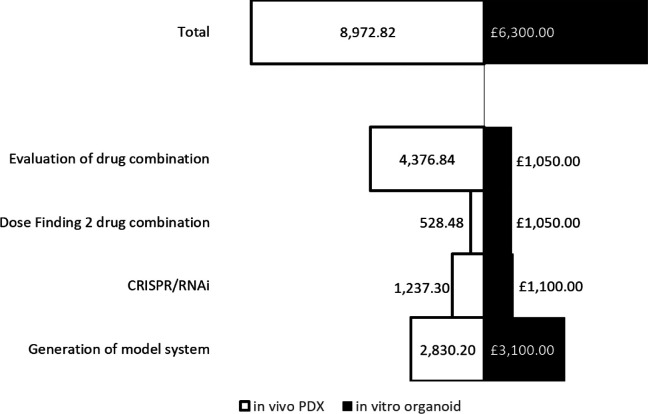
A comparative costs analysis between 3D *ex vivo* and *in vivo* preclinical models show an approximate 30% costs benefit and consequently increased financial sustainability when using *ex vivo* organoid models.

ALL blasts from three different clinical samples were co-cultured with BM-MSC in routine 2D cultures and with 3D spheroids. Cell proliferation was monitored over a 5 day period through tryphan blue cell counts. We observed that when co-cultured with BM-MSC-spheroids the blasts showed 2-fold higher cell proliferation compared to 2D co-cultures ( Figure 4a- c). Next, we repeated the co-cultures under treatment with the steroid Dexamethasone which is used to treat ALL. We observed that blasts showed marked reduction in sensitivity to Dexamethasone in 3D MSC organoid co-cultures compared to 2D co-cultures ( Figure 4d- f). These data show the advantage of 3D organoid based co-culture systems over routine 2D cultures in two respects: 1. Achieving blast cycling which is essential for the action or testing of majority of anti-cancer treatments 2. Modelling treatment resistance in the laboratory – a major current day clinical challenge in cancer management. Finally, we perform a costs analysis and show improved financial sustainability in our *ex vivo* organoid platform compared to animal models ( Figure 5).

## Discussion

Relapse is still a major concern within the treatment of leukaemia, relapse disease is often chemo resistant, meaning many therapies cease working in patients with relapse disease leading to an increased mortality. ^3^ Differing cytogenic abnormalities in this disease are known and patient stratification and precision medicine are implemented in an effort to treat individual disease in the most effective way possible, allowing weaknesses in tumour biology to be exploited therapeutically. However, high drug attrition rates remain a challenge when developing such improved treatments in ALL. High drug attrition has been attributed to preclinical models not being clinically translatable. Key barriers hindering bench to bedside translation when using complex *in vivo* models include species specificity, high financial costs, and lengthy experiments. Patient samples do not always engraft in mice. Samples that do engraft form successful xenograft models in 3-6 months at which point drug testing can be started which takes another 3-4 weeks.

A 3D BM-mesenchyme-leukaemia spheroid model will provide researchers with a biomimetic platform where clinical samples can be cultured within the context of their microenvironment, and patient specific drug testing performed within a week. Besides delivering drug response data within clinically relevant timeframes the relative simplicity of *ex vivo* spheroid models mean that they are tractable, transferrable and sustainable. Consequently, such models have wide applications in translational research in academia and industry alike. These models can be set up in laboratories and SMEs locally, nationally and globally that do not contain infrastructure for animal procedures. Following further extensive validation, such spheroid biobanks also have the potential to be embedded within clinical trials and healthcare systems for the purposes of detecting responders as well as to aid risk stratification.

In this paper we show that our 2D model itself brought about significant animal replacement locally. Leukaemia research at Newcastle requires 3600 animal procedures every year. Local metrics confirmed that using the 2D pilot approach we replaced mice in 67 drug tests last year. Given minimum of 40 animals are needed per drug test we replaced 2680 animals (out of 3600) which constituted a minimum 73% local animal replacement. To improve biomimicry and consequently translatability and transferability of our model, we develop 3D BM-mesenchyme spheroids. We show that these 3D spheroids show superior ability in supporting culture of leukaemia patient samples. We also show sensitivity of leukaemia cells to drugs such as dexamethasone is reduced when these cells are being co-cultured with 3D spheroids. This means that compared to 2D MSC cultures, MSC spheroids provide superior protection to leukaemia cells against dexamethasone treatment. are appropriate in modelling treatment resistance which remains a major clinical challenge in cancer management. This simple and tractable 3D prototype will form the first steppingstone in developing next generation 3D preclinical models with improved *in vivo* and patient drug response predictability. Such sustainable *ex vivo* models will ultimately replace existing moderate severity procedures in leukaemia research with models that successfully impact clinical outcome.

## Data Availability

Mendeley Data. A human mesenchymal spheroid prototype to replace moderate severity animal procedures in leukaemia drug testing. https://doi.org/10.17632/56npmkbpfb.1. ^24^ This project contains the following underlying data:
- 3D spheres.tif - 3D Sphere.tif - drug combination.xlsx -
*in vivo* Vs *in vitro* costs.xlsx - Raw Figure Data.xlsx Data are available under the terms of the Creative Commons Attribution 4.0 International license (CC-BY 4.0).
