# A human mesenchymal spheroid prototype to replace moderate severity animal procedures in leukaemia drug testing

**DOI:** 10.12688/f1000research.123084.2

**Published:** 2023-11-06

**Authors:** Aaron Wilson, Sean Hockney, Jessica Parker, Sharon Angel, Helen Blair, Deepali Pal

**Affiliations:** 1 Wolfson Childhood Cancer Research Centre, Translational and Clinical Research Institute, Newcastle University, UK, Newcastle upon Tyne, NE1 7RU, UK; 2 Applied Sciences, Northumbria University, Newcastle upon Tyne, NE1 8ST, UK

**Keywords:** Animal replacement, 3D models, Preclinical models, cancer research, leukaemia

## Abstract

Patient derived xenograft (PDX) models are regarded as gold standard preclinical models in leukaemia research, especially in testing new drug combinations where typically 45-50 mice are used per assay. 9000 animal experiments are performed annually in the UK in leukaemia research with these expensive procedures being classed as moderate severity, meaning they cause significant pain, suffering and visible distress to animal’s state. Furthermore, not all clinical leukaemia samples engraft and when they do data turnaround time can be between 6-12 months. Heavy dependence on animal models is because clinical leukaemia samples do not proliferate *in vitro.* Alternative cell line models though popular for drug testing are not biomimetic – they are not dependent on the microenvironment for survival, growth and treatment response and being derived from relapse samples they do not capture the molecular complexity observed at disease presentation. Here we have developed an *in vitro* platform to rapidly establish co-cultures of patient-derived leukaemia cells with 3D bone marrow mesenchyme spheroids, BM-MSC-spheroids.  We optimise protocols for developing MSC-spheroid leukaemia co-culture using clinical samples and deliver drug response data within a week. Using three patient samples representing distinct cytogenetics we show that patient-derived-leukaemia cells show enhanced proliferation when co-cultured with MSC-spheroids. In addition, MSC-spheroids provided improved protection against treatment. This makes our spheroids suitable to model treatment resistance – a major hurdle in current day cancer management

Given this 3Rs approach is 12 months faster (in delivering clinical data), is a human cell-based biomimetic model and uses 45-50 fewer animals/drug-response assay the anticipated target end-users would include academia and pharmaceutical industry. This animal replacement prototype would facilitate clinically translatable research to be performed with greater ethical, social and financial sustainability.

Research highlights **Scientific Benefit**

A 3D spheroid-based approach with improved clinical relevance for *ex vivo* co-culture of patient-derived-leukaemia samples.

**3Rs Benefit**

To replace animal experiments, including moderate severity animal (mice) procedures in leukaemia research and drug testing.

**Practical Benefit**

A 3Rs approach that yields drug response data quickly and is ethically, socially and financially more sustainable than its *in vivo* counterparts.

**Current applications**

Exploration of leukaemia biology such as stem/progenitor proliferation, stem/progenitor-niche interactions, niche-impacted treatment resistance and to obtain drug response data.

**Potential applications**

Adapt the approach to include other haematological cancers as well as bone cancers.

## Introduction

Acute lymphoblastic leukaemia (ALL) management in children is hindered by two chief obstacles: 1. Treatment toxicity ^1^ ^,^ ^2^ 2. 15-20% of patients go into relapse, when the disease can become treatment resistant. ^3^ This highlights the requirement for improved treatments. High drug attrition rates remain a major hindrance to anti-cancer drug development. 95% of de novo drugs entering phase 1 trials never reach patients. ^4^ ^–^ ^6^ Three key improvements ^5^ have been identified to reduce attrition rates: 1. Increase in scientific collaborations 2. Tumour biology driven preclinical studies and clinical trials 3. Improved development of translatable preclinical cancer models that would screen out “false positives” therapeutics before these reach clinical trials. ^5^

The gold standard preclinical model in leukaemia research is a PDX model. ^7^ ^–^ ^9^ Recent Home Office annual returns show that 195,407 mice are used annually in cancer research. Indeed, cancer research remains the most common applied sciences area to use animal procedures. Given leukaemia research is heavily dependent on mouse models combining these metrics with financial data from major cancer funders in the UK suggest that 5% of these mice or at least 9000 mice are used in leukaemia research every year. Although leukaemia is a very aggressive disease, leukaemia cells do not maintain viability outside the body. ^9^ Several research groups would likely use primary patient for *in vitro* studies before moving into *in vivo* models, nevertheless *in vivo* models are still used as key models to expand primary samples as vital research resources. This causes heavy reliance on PDX models, where leukaemia cells are engrafted into immunocompromised mice to amplify patient-derived leukaemia cells, to study cancer biology and perform translational studies. ^7^ ^,^ ^8^ Patient-derived leukaemia cells can take 2-12 months to engraft in mice before any preclinical drug testing can begin. Furthermore, most of these procedures involve mice developing significant adverse effects such as weight loss, fur ruffling, back hunching as well as increased white cell counts.

The pipeline for preclinical testing using mouse PDX models is: 1. Toxicity analysis to determine no observed adverse effect limit (NOAEL)/maximum tolerated dose (MTD): 15-20 mice per drug. ^9^ 2. Pharmacokinetic studies to determine drug maximum plasma/tissue levels and kinetics of drug clearance:15-30 mice per drug. ^10^ 3. Drug efficacy studies and pharmacodynamics assays on tumour cell response: 5-10 mice needed per drug treatment group. ^11^ Per drug group a total of 40-50 animals are needed for single drug assays and twice as many for drug combination assays. ^9^ ^–^ ^11^ Moreover, *in vitro* cancer models preceding *in vivo* validation experiments rely on cell lines, which retain cytogenic characteristics of leukaemia. However being derived from patients with relapsed disease these samples do not represent the molecular complexity of disease that is observed at presentation. ^12^ ^,^ ^13^ Cell lines have furthermore adapted to grow *in vitro* as suspension monocultures, and therefore do not represent cancer within the context of its microenvironment. Therefore, these models show limited *in vivo* predictivity, leading to increase *in vitro* to *in vivo* drug attrition rates.

ALL is a disease primarily of the bone marrow (BM), the BM comprises cell types including haematopoietic, mesenchymal stroma cells (MSC), osteoblasts, osteoclasts, endothelia, perivascular and immune cells. Difficulty in culturing patient derived ALL cells *ex vivo* alludes to the vital role that the BM-niche plays in cancer biology. The leukaemia BM-microenvironment plays a key role in disease, mediating cell-cell signalling and conferring treatment protection to leukaemia cells. ^14^ ^,^ ^15^ Mesenchyme-leukaemia interactions in the BM generate two leukaemia subpopulations, slow cycling, dormant- and actively cycling, disease propagating- populations. ^12^ ^,^ ^16^ Many treatments are effective in reducing active cycling leukaemia cells; however, dormant leukaemia-populations can survive treatment, seeking refuge within the BM-microenvironment. These dormant cells can later, post treatment, shift back into a cycling state, leading to disease relapse ^13^ ^,^ ^16^ and therefore capturing dormant ALL cells *in vitro* is important. Furthermore, targeting leukaemia-niche interactions may present as promising therapeutic approaches. We have developed *in vitro* 2D co-culture platforms in previous studies ^12^ ^,^ ^17^ to 1. conduct preclinical drug screening using patient-derived leukaemia cells, thereby reducing dependence on cell lines, consequently minimising *in vitro* to *in vivo* drug attrition rates and 2. explore the impact of human BM cells in driving ALL biology such as leukaemia proliferation, dormancy and treatment resistance. ^12^ Furthermore, to build proof-of-confidence in application data, we previously showed that such human relevant *ex vivo* platforms have potential to detect therapeutically exploitable targets that disrupt leukaemia-BM cell interactions conferring treatment resistance to the cancer cells. ^12^ ^,^ ^18^ Such models also have greater tractability than *in vivo* procedures, further mitigating reliance on mouse experiments to perform mechanistic studies. However, standard 2D models, including co-cultures, do not sufficiently portray functional biomimicry and do not recapitulate structural intricacies of the leukemic niche. Nor do such 2D models capture the complex polarity of cell-cell interactions ^19^ that are likely to influence leukaemia cell growth.

Recent advances in medicine have embraced 3D models as miniature *in vitro* representations of specific organs. ^20^ 3D models have been proven to be useful models of human disease, particularly in cancer research. ^21^ Modern medicine is embedding the use of organ-on-a-chip systems which have led to advances in liver and lung drug screening, providing more accurate toxicological reports than 2D systems. ^20^ The complex and dynamic nature of ALL and the BM mesenchymal microenvironment highlights the need for a more representative drug screening platform. This paper aims to do this by developing mesenchymal spheroids, to support the co-culture of patient derived leukaemia samples, consequently enabling stratified-medicine and precision oncology driven therapeutic investigations. ^22^

## Methods and materials

### Materials

**Table 1.** Materials used.

Material	Manufacturer	Catalogue Identifier
**General**		
Trypsin-EDTA Solution 10X	Sigma-Aldrich, Dorset, UK	59418C
Phosphate-Buffered Saline	Thermo Fisher Scientific, Hertfordshire, UK	10010023
Gentle Cell Dissociation Reagent	Stem Cell Technologies, Stem Cell, UK	**100-0485**
Heat Inactivated Foetal Bovine Serum	Thermo Fisher Scientific, Hertfordshire, UK	10500064
Matrigel HESC-Qualified Matrix	Corning, Kennebunk, USA	CLS354277
Low-glucose Dulbecco's Modified Eagle's Medium	Sigma-Aldrich, Dorset, UK	D5546
**Cells**		
Primary Mesenchymal Stem Cells	Obtained from patients undergoing total hip replacement in view of osteoarthritis ^17^	
PDX sample L707	Obtained from patient with E2A-HLF translocation ^12^ ^,^ ^17^	
PDX sample L49120	Obtained from patient with BCR-ABL translocation ^23^	
PDX sample MS40	Obtained from patient with MLL rearrangement ^24^	
**MSC culture media**		
Low-glucose Dulbecco's Modified Eagle's Medium	Sigma-Aldrich, Dorset, UK	D5546
Heat Inactivated Foetal Bovine Serum	Thermo Fisher Scientific, Hertfordshire, UK	10500064
Penicillin/Streptomycin	Sigma-Aldrich, Dorset, UK	P4333
Basic FGF	Sigma-Aldrich, Dorset, UK	PHG0024
L-Glutamine	Sigma-Aldrich, Dorset, UK	59202C
Matrigel HESC-Qualified Matrix	Corning, Kennebunk, USA	CLS354277
**Leukaemia Blast Co-Culture**		
SFEM, Serum-Free Medium for Culture and Expansion	Stem Cell Technologies, Stem Cell, UK	09650
**Organoid formation**		
AggreWellM 400 24-well Plate	Stem Cell Technologies, Cambridge, UK	34411
AggreWell Rinsing Solution	Stem Cell Technologies, Cambridge, UK	**07010**
Costar Ultra-low Attachment 6 Well Plate	Corning, Kennebunk, USA	3471
**Real-Time PCR Analysis**		
QIAShredder	Qiagen, Manchester, UK	79656
Beta-mercaptoethanol	Sigma-Aldrich, Dorset, UK	M6250
RNEasy Mini Kit	Qiagen, Manchester, UK	74104
RNase-Free DNase Set	Qiagen, Manchester, UK	79254
**Primer Sequences**		
CDH2 forward primer, Sigma-Aldrich, Dorset, UK	GGTGGAGGAGAAGAAGACCAG	OLIGO
CDH2 reverse primer, Sigma-Aldrich, Dorset, UK	GGCATCAGGCTCCACAGT	OLIGO
CD90 forward primer, Sigma-Aldrich, Dorset, UK	CACACATACCGCTCCCGAAC C	OLIGO
CD90 reverse primer, Sigma-Aldrich, Dorset, UK	GCTGATGCCCTCACACTT	OLIGO
NES forward primer, Sigma-Aldrich, Dorset, UK	AGAGGGGAATTCCTGGAG	OLIGO
NES reverse primer, Sigma-Aldrich, Dorset, UK	CTGAGGACCAGGACTCTCTA	OLIGO
GAPDH forward primer, Sigma-Aldrich, Dorset, UK	GAAGGTGAAGGTCGGAGTC	OLIGO
GAPDH reverse primer, Sigma-Aldrich, Dorset, UK	GAAGATGGTGATGGGATTTC	OLIGO
**Staining Reagents**		
Calcein AM	BioLegend	425201
Hoechst 33342	MERCK, Sigma Aldrich	
PBS	Gibco	15400-054
FITC CD105 Antibody	BioLegend	323203
AlexaFluor 700 CD73 Antibody	BioLegend	344041
Brilliant VioletTHY1 Antibody	BioLegend	328121

### Methods

***Ethical approval***

Patient leukaemia cells were obtained from the Newcastle Biobank (REC reference number 07/H0906/109+5). All samples were acquired following written informed consent. Animal data shown here reflect unpublished data that were existing from past experiments. These experiments were carried out as per previously published methods. ^12^ ^,^ ^17^ ^,^ ^25^ PDX-ALL samples used in this project are from our cryopreserved PDX bank where the samples were generated in previous/earlier projects. All animal studies were performed in accordance with UK Animals (Scientific Procedures) Act, 1986 under project licence P74687DB5 following approval of Newcastle University animal ethical review body (AWERB).

***2D BM-MSC-ALL co-culture***

Patient derived PDX-ALL samples were cultured on 2D monolayers of MSC cells using a protocol published in an earlier study. ^17^ MSC used in this study are between passages ranging from p2 – p5. MSC were seeded on Matrigel coated 48 well plates at 1×10 ^4^ cells/0.5 mL/cm ^2^ in their respective media (MSC media, Table 1). After 24 hours, the media was aspirated, and the cells rinsed with 1 mL SFEM. Leukaemia cells were then plated onto the MSC layers, at a density of 0.25×10 ^5^ cells/1 mL per well, suspended in SFEM media.

***3D culture of BM-MSC***

MSC were transferred to a 6-well low adhesion plate and cultured in 2 mL MSC media ( Table 1) for 48 hours, after which, MSC spheroids were seen to form. Spheroids obtained using this method were irregular in shape and therefore experiments were repeated and optimised using Aggrewell400 plates. Aggrewell plates were prepared by rinsing each well with 0.5 mL of Aggrewell rinsing solution, spinning at 1500 g for 5 minutes and then aspirating excess solution. MSC were seeded at varying concentrations to track effect of seeding density on spheroid size. Cells were trypsinised and seeded in single cell format on Aggrewell plates at varying seeding densities in MSC media. Aggrewell plates were spun at 100g for 3 minutes to ensure all cells reached the bottom of the microwells. After 48 hours, MSC spheroids were ready for harvest and manually transferred onto low adhesion plates. Using a stereomicroscope fitted within a Class II biological safety cabinet, the spheroids were transferred onto respective culture-ware using a Gilson p1000 pipette with the tip-end cut off using sterile scissors.

***3D BM-MSC-ALL co-culture***

Leukaemia cells from PDX-ALL samples were co-cultured with the MSC spheres in SFEM media, at 2×10 ^5^ cells/mL, in 5 ml final volume, using low adhesion 48 well plates over a 5-day period. On day of harvest, the leukaemia cells and MSC spheroids were transferred into a 30 ml universal tube. This was centrifuged at 1500 RPM for 5 minutes. After aspirating the supernatant, 0.5 ml of X1 Trypsin EDTA was added and cultures incubated at 37C, 5% CO _2_ for 2 minutes. Cells were mechanically dissociated into single cells by resuspending with a pipette. 4 ml of FBS was added and this was centrifuged at 1500 RPM for 5 minutes. Supernatant was aspirated and cells were resuspended in 1ml of fresh SFEM media. During cell count leukaemia cells were distinguished from MSC based on their morphology and size difference, the MSC are 3-4 times the size of leukaemia cells.

***Dexamethasone 3D drug assay***

10 million leukaemia cells were spun at 500g X 5 minutes and plated onto 3D MSC spheres in 48 well plates as described above. 5 nM dexamethasone (stock dissolved in methanol to generate 10 mM dexamethasone concentration, working concentration of dexamethasone was made by performing serial dilutions using SFEM media) was added to desired wells and leukaemia cells were cultured for 5 days. Experiments were repeated to include a total of 3 biological replicates with each replicate including 3 independent experiments.

***3D Spheroid Calcein AM/Hoechst-33342 staining***

MSC were seeded into Aggrewell plates to obtain a final spheroid size of 200 cells per microwell. Cells were cultured in the Aggrewell format for 3 and 7 days following which resultant spheroids were collected for the staining procedure. The calcein stain was prepared by reconstituting the lyophilized reagent to a 1mM stock in DMSO. From this, 1 μM working solution was prepared by diluting into sterile calcium free PBS. The MSC spheres were resuspended in PBS at a density of approximately 1×10 ^7^cells/ml, to which 10 μl of 1 μM calcein stain was added to final concentration of 0.01 μM. Cells were incubated at 37°C for 20 minutes in the dark. Cells were pelleted and resuspended in pre warmed media and left for 10 minutes to ensure optimal calcein retention before imaging. Samples that were double stained with Hoechst-33342 were stained for calcein as above and following the 10 minute incubation, were stained with Hoechst-33342 at final working concentration of 10 μg/ml in pre warmed media for 20 minutes. Digital Images were captured using Leica Application Suite X (LASX) version 3.5.7.23225.

***MSC Marker Antibody staining***

MSC Spheroids were collected from Aggrewell plates, disrupted to single cells using a trypsin EDTA wash followed by gentle mixing. Remaining cell clumps were removed by passing sample through a 40 μm PluriStrainer. Cells were counted using a trypan blue exclusion assay. 1 million cells were resuspended in PBS to wash before blocking in 10%FBS/PBS. Cells were again spun down and resuspended in 200 μl PBS. 5 μl antibody was added and samples were incubated at room temperature for 1 hour in the dark. Following incubation, 800 μl 10% FBS/PBS was added and cells were spun down again before being resuspended in PBS for running on flow cytometer.

***Extraction of mRNA***

RNA was purified from cell pellets (viable cells centrifuged at 5000 g X 5 minutes followed by aspiration of supernatant media) using the RNeasy mini kit, and as per manufacturer protocols. The RNA yield and quality was checked using the nanodrop ND-1000 spectrophotometer.

***cDNA synthesis***

RevertAidTMH Minus First Strand cDNA Synthesis Kit was used to synthesise cDNA from the RNA isolated. 500 ng RNA was collected and added to RNase/DEPC free water to a final volume of 11 μl. 1 μl (dN)6 (200 mg/l) random hexamers was added, mixed gently by inverting the vial and briefly centrifuged. Using a GeneAmp PCR system 2700 the sample was incubated at 65°C for 5 minutes, after which the sample was immediately placed on ice, 8 μl of the master mix ( Table 2) was added, the samples were vortexed and briefly centrifuged. The samples were placed back in the PCR machine to incubate at 25°C for 10 minutes, 42°C for 60 minutes and 75°C for 10 minutes to terminate the reaction. The product was either used immediately for RT-PCR or stored at -20°C for short term storage.

**Table 2.** cDNA master mix constituents.

Reagents	Volume (μl)
5x Reaction Buffer	4
RNase Inhibitor	1
10mM dNTP	2
RevertAid H Minus MMLV RT	1
**Total Volume**	**8**

***qRT-PCR***

Upon receipt, primers were reconstituted in RNase/DNase free water to a final stock concentration of 100 μM. PCR Mix ( Table 3) was loaded onto PCR plate in triplicates. The plate was sealed and centrifuged for 1 minute at 1000 RPM and placed in an applied Biosystems 7900HT Sequence Detection System. ViiA7TM System was used to run the qRT-PCR plate and cycle threshold (Ct) and melting curves were obtained for analysis.

**Table 3.** qRT-PCR mix constituents.

Reagents	Volume (μl)
SYBR Green	5
10μM Working Primer	0.3
RNase-free water	2.7
cDNA Sample	2
**Total**	**10**

***Costings analysis of in vivo versus in vitro models***

Costings of *in vitro* models were estimated using list prices of media, reagents and other materials associated with developing and using the models for a 2 drug combination dose finding assay. This included 5 biological replicates, 3 experimental repeats with each having 3 technical repeats with a total number of experiments of 45. These costs were divided into the following categories: Production of clinical/patient-derived leukaemia samples; *in vitro* CRISPR/RNAi organoid generation; Dose/IC50 finding for 2 drug combination; and evaluation of drug combination.

*In vivo* costings were calculated using figures obtained from the Comparative Biology Centre, Newcastle University. This included fixed costs including mouse purchase from an NSG in-house colony, housing and IVIS use. Comparative *in vivo* equivalents to the *in vitro* design accounted for: production of PDX engrafted tissue (5 PDX, 6 NSG mice/PDX); *in vivo* CRISPR/RNAi (control and treatment groups, 5 NSG mice each); Dose finding for 2 drug combination (3 mice per sex); and evaluation of the combination (4 arms, 6 mice per arm). All total costings were calculated using Microsoft Excel and figures were created using Microsoft Excel.

***Data analysis***

Microscopy images were analysed using the Windows download bundled with Java 8. Version 1.4.3. Real time qPCR data were captured and analysed using the StepOne Plus Real-Time PCR System (ThermoFisher Catalog No: 4376600) and Software package StepOne Software v23.

## Results

### Development and validation of BM-MSC spheres

**Figure 1.**
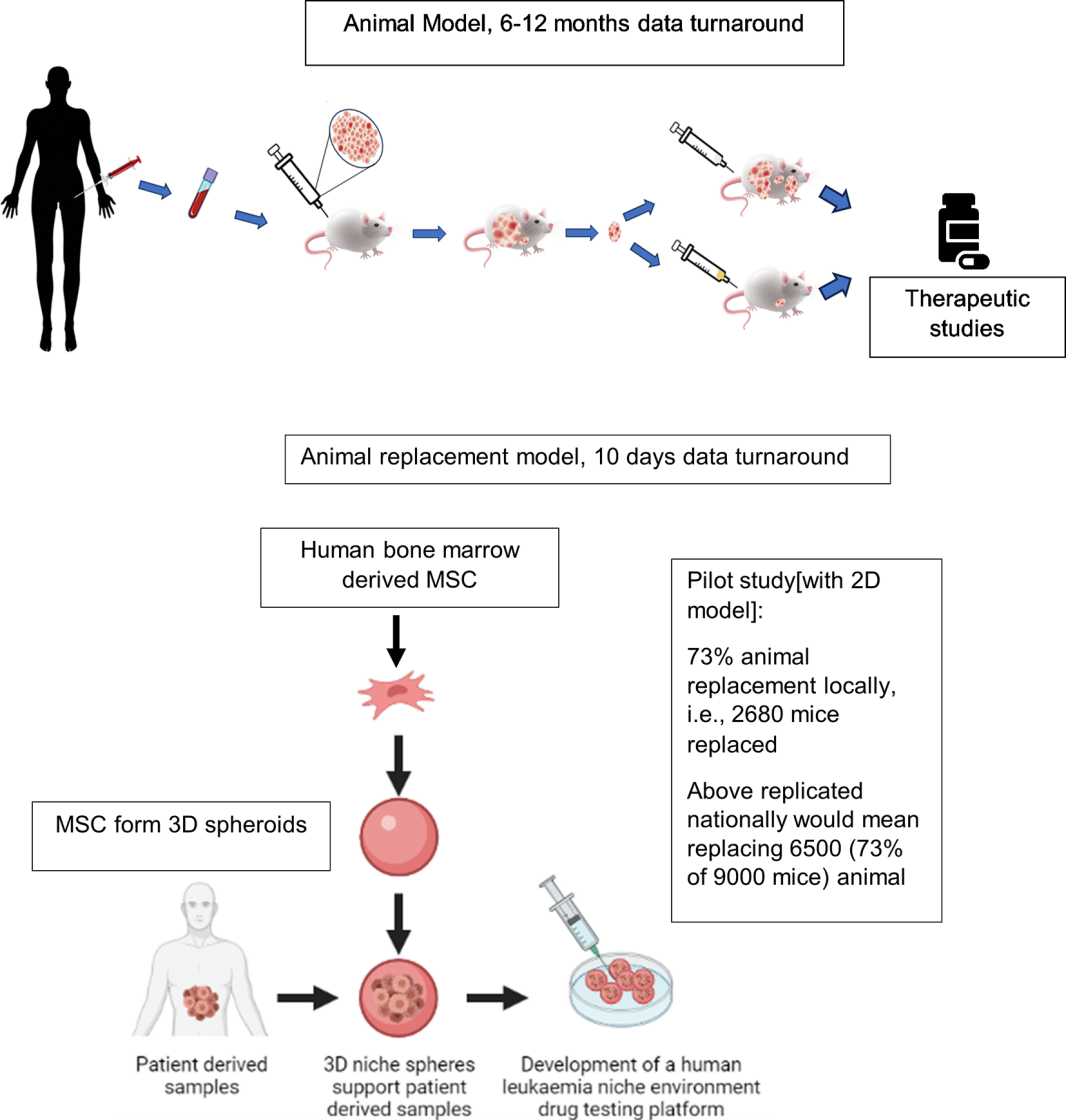
Comparison of existing *in vivo* preclinical leukaemia models with newly developed *ex vivo* animal replacement organoid models. Key benefits of the animal replacement organoid models include species specificity and consequently higher biomimicry.

**Figure 2.**
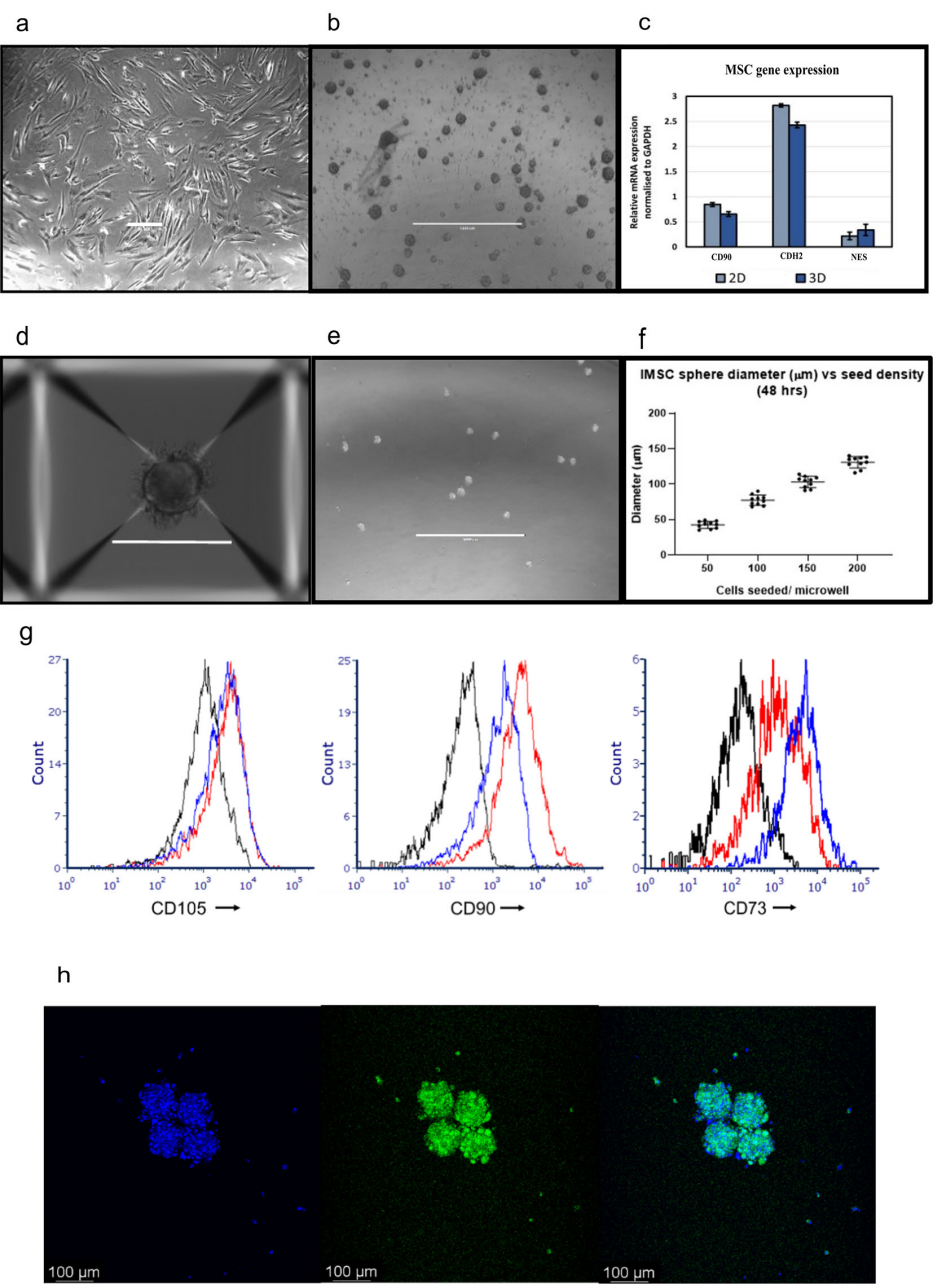
Aggrewell plates generate uniform 3D spheroids from single cell suspension of the desired cell-type. SD from three independent experiments (using MSC from same patient) have been plotted as error bars. Phase contrast photographs showing (a) 2D MSC, scale bar = 100 μM and (b) 3D MSC, scale bar = 200 μM after 96 hours of growth. (c) Mesenchymal marker levels quantified through qPCR and normalised to GAPDH. (d,e) Phase contrast photograph of MSC spheroids generated through culture using Aggrewell plates. Scale bar = 1000 μM (f) Correlation between cell seeding densities and size of resultant spheroids. Data captured following 48 hours of seeding. (g) CD105, CD90 and CD73 expression in MSC. Black = unstained. Blue = 2D MSC. Red = 3D MSC. (h) Hoechst-33342-Calcein staining in 100 μM spheroids, taken at day five of 3D culture. Calcein (green) stains live cells, Hoechst-33342 (blue) stains live and dead cells.

We find that 2D *ex vivo* human cell-based co-culture platforms potentially enable 73% (metrics obtained locally) replacement of animals in drug testing experiments. Drug testing experiments are conventionally carried out using PDX-mouse-models, currently regarded as gold standard preclinical leukaemia models ( Figure 1). Here we develop 3D BM-MSC-spheroids, to improve the spatial biomimicry of functional cell-cell contacts, by subculturing MSC onto low adhesion non-tissue culture treated plates ( Figure 2a-b). We show that 3D MSC retain expression of key genes such as THY1 (CD90), CDH2 and NES ( Figure 2c).

Aggrewell400 plates are designed for higher throughput spheroid engineering. These plates further aid the production of MSC spheres that show greater consistency in shape and size. Each well on these plates house 400 conical microwells, funnelling single cells and encouraging them to conglomerate into spheroids ( Figure 2d). Here we use these microwells to create uniform BM-MSC spheres ( Figure 2e), consequently allowing standardisation of the 3D technique. We find a distinct correlation between seeding cell density and size of spheroids, with higher seeding densities resulting in generation of more uniformly sized spheroids ( Figure 2f). For subsequent experiments, we use MSC spheroids of diameter 100 μM size, generated with 200 MSC seeded per microwell. In addition, we show that MSC harvested from these 3D spheroids retain the expression of MSC markers CD105, CD90 and CD73 at protein level ( Figure 2g). We furthermore confirm the retention of cell viability within these spheres via Hoechst-33342 dye, which stains live and dead cells and calcein dye, which stains live cells ( Figure 2h).

### Patient-derived ALL cells co-cultured with BM-MSC show superior proliferation and reduced dexamethasone sensitivity

**Figure 3.**
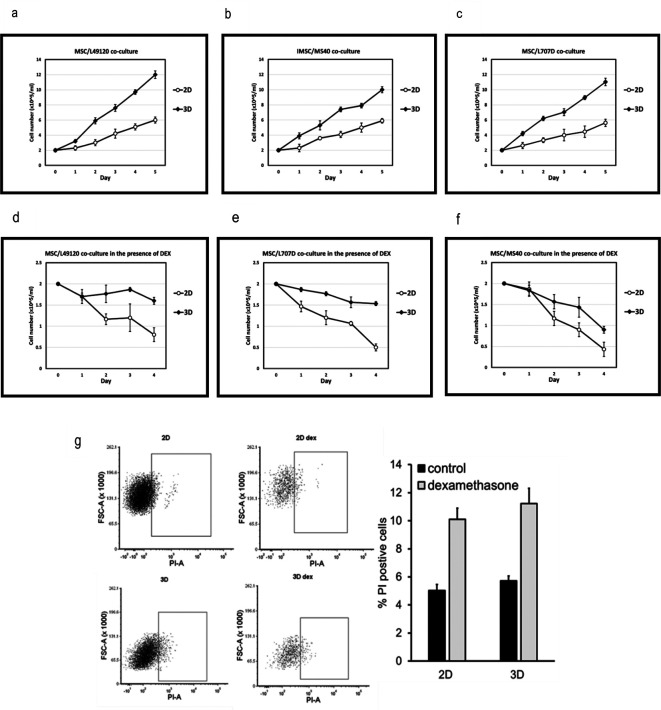
3D MSC-spheroids confer higher proliferation and improved survival onto patient-derived ALL. Growth kinetics, measured via viable cell counts of ALL samples (a,d) L49120, (b,e) MS40 and (c,f) L707D across a 5 day period, with and without low dose dexamethasone (10nM) pressure, as co-cultures with 3D MSC spheres versus 2D MSC-co-culture. L49120, MS40 and L707D refer to three different patient samples, i.e., 3 biological repeats. (d,e,f) Dexamethasone treatment, data shown for 5nM treatment, is less effective in a 3D microenvironment. N= 3. (g) Flow cytometry data, representative plots, showing propidium iodide, PI, positive ALL cells (sample L707) following five days of 10nM dexamethasone treatment. The column graph shows % ALL cells stained positive for PI. N = 2.

We co-culture PDX-ALL cells with 2D MSC or with 3D MSC-spheroids, and monitor ALL cell counts over a 5-day period. We find that when co-cultured with MSC-spheroids, the leukaemia cells show 2-fold higher cell proliferation compared to 2D MSC-co-cultures ( Figure 3a-c). Next, we repeat the co-cultures under low dose dexamethasone (5nM) treatment pressure. Dexamethasone is a glucocorticoid routinely used in the clinics to treat ALL. We find that ALL cells show greater reduction in sensitivity to dexamethasone when co-cultured with 3D BM-MSC ( Figure 3d-f). This could potentially be due to improved cell-cell contacts and physiologically relevant cell polarity being achieved in a 3D format. This might have caused increased 3D versus 2D MSC-conferred ALL proliferation and/or improved ALL survival. Reduced sensitivity could also be due to altered drug availability within 3D spheroids. These data highlight the advantage of 3D organoid-based co-culture systems over routine 2D cultures in two respects: 1. Achieving higher ALL cell cycling which is essential for the action or testing of majority of anti-cancer treatments 2. Reduced drug sensitivity, possibly owing to improved PDX-ALL survival via MSC-conferred treatment protection. We further investigate the ability of MSC-spheroids to reduce ALL cell death and find that when treated with a higher dose of dexamethasone, that is, 10nM, then both 2D and 3D MSC-spheroids support survival of leukaemia cells ( Figure 3g). We now progress to reveal proof-of-confidence-in-application data, justifying the suitability of our platform in conducting combinatorial drug testing.

### Co-cultures of 3D BM-MSC with PDX-ALL cells how high *in vivo* predictivity

**Figure 4.**
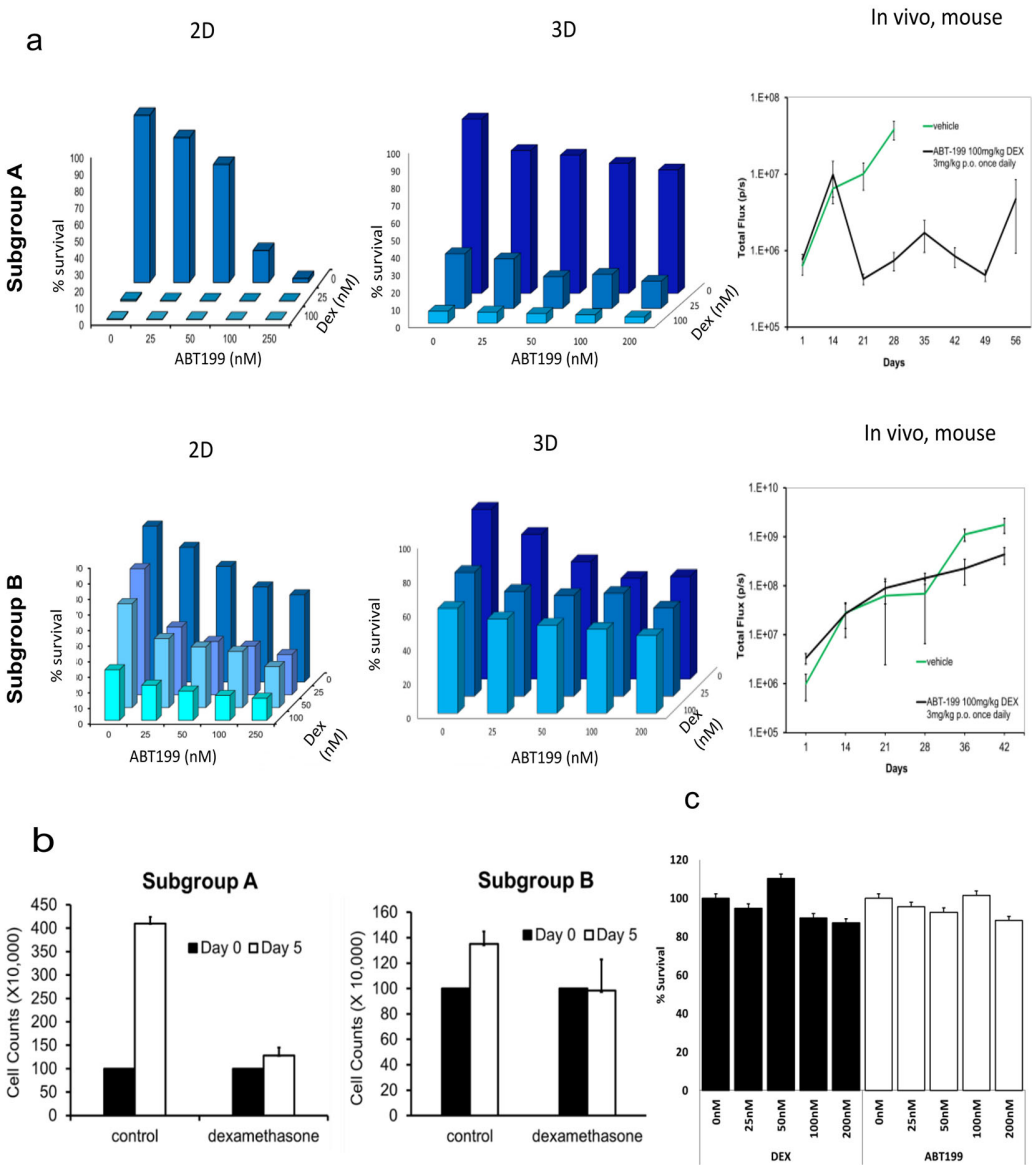
*In vitro* co-culture platform delivers drug response data with high *in vivo* predictivity to minimise *in vitro* to *in vivo* drug attrition rates. Leukaemia cells from leukaemia cytogenetic subgroup A is a responder to ABT199 (targets BCL2 in BCL2 positive ALL) and dexamethasone drug combination *in vitro* and *in vivo.* On the other hand, leukaemia cells from subgroup B show low sensitivity to this drug combination *in vitro* in both 2D and 3D formats, and subsequently poor response is also noted *in vivo* using PDX mice models. *In vitro* data readout includes viable cell counts; *in vivo* data (right hand graphs) is shown as bioluminescence imaging from luciferase expressing PDX cells in total flux(p/s). Total flux is a surrogate of blast number *in vivo.* Error bars shown refer to standard error (SE) using 4 mice per treatment group. Dexamethasone and ABT-199 are used in the clinics to treat ALL. b. ALL cell counts on day 0 versus day 5 of culture, with and without treatment with 25 nM dexamethasone. N = 3. Error bars = SD. c. Cell counts following dexamethasone and ABT-199 treatment of 3D MSC.

**Figure 5.**
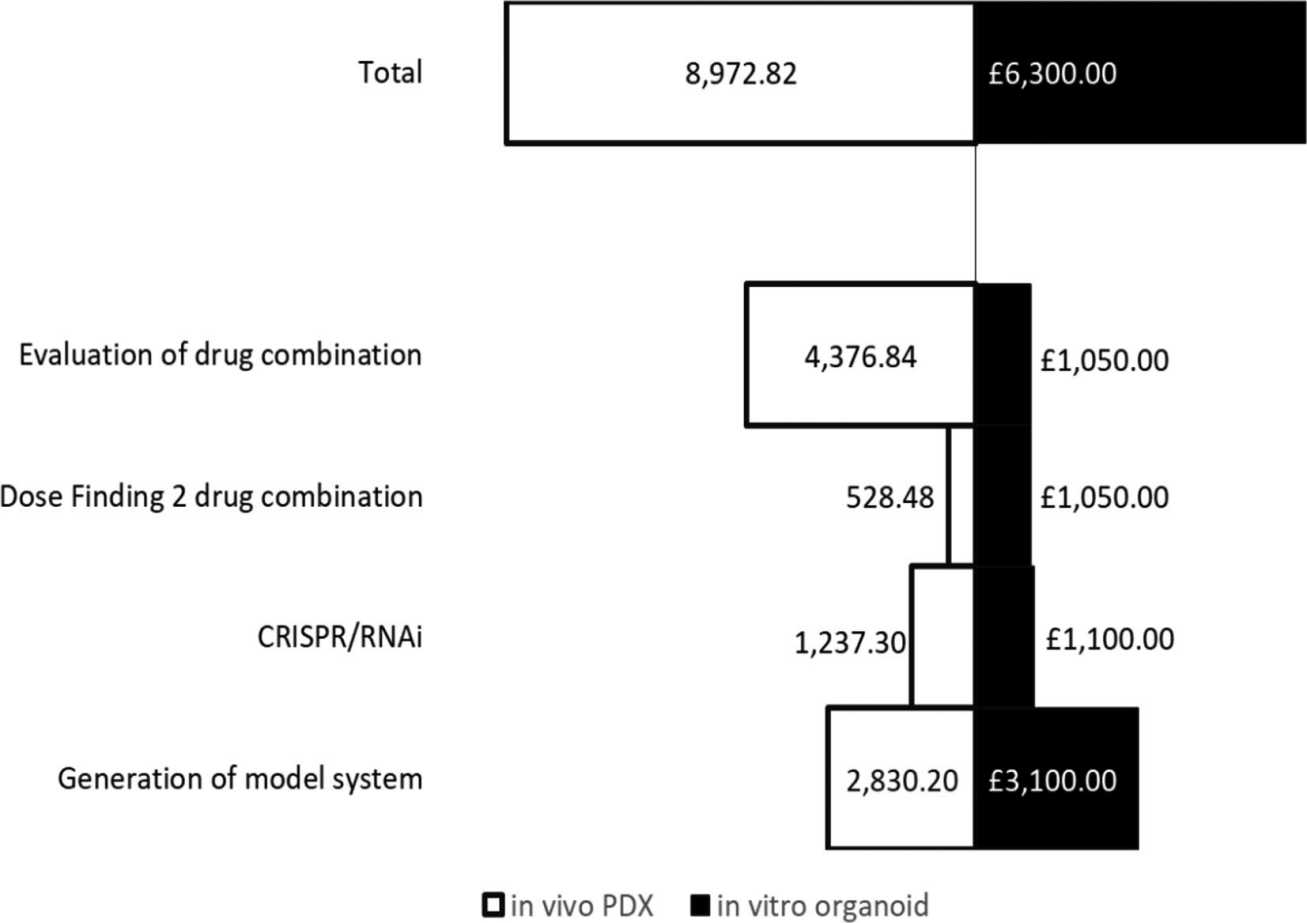
A comparative costs analysis between 3D *ex vivo* and *in vivo* preclinical models show an approximate 30% costs benefit and consequently increased financial sustainability when using *ex vivo* organoid models.

Improved *in vivo* predictivity is a key requisite for *ex vivo* animal replacement models, if these platforms are to effectively replace mouse experiments. We confirm that BM-MSC/leukaemia 2D co-culture platform generate drug dose response data that was largely comparable with drug response observed *in vivo* in PDX-mouse models ( Figure 4a). Furthermore, we reveal that when PDX-ALL cells are co-cultured with 3D BM-MSC-spheroids they show greater reduction in sensitivity, compared to the 2D co-culture arm, against dexamethasone, ABT-199 (a molecularly targeted therapy against BCL-2 positive blood cancers), and dexamethasone-ABT-199 combination in a sample subgroup that exhibited reduced *in vivo* efficacy (obtained from previously unpublished *in vivo* data existing in the lab from earlier studies). This shows the ability of our 3D co-culture model to screen off false-positive hits derived from 2D models, consequently minimising *in vitro* to *in vivo* attrition rates. We further confirm that when co-cultured with 3D MSC spheres, ALL cells expectedly proliferate in 3D-MSC spheroid-co-cultures, however, fail to increase in cell counts on day five, in the dexamethasone treatment arm ( Figure 4b). Moreover, we show that neither dexamethasone, nor ABT-199, have any effect on survival of MSC, when treated for five days within a 3D sphere format ( Figure 4c). Finally, we perform a costs analysis and show improved financial sustainability, namely a 30% costs benefit, in our *ex vivo* organoid platform compared to animal models ( Figure 5).

## Discussion

Patient stratification and precision oncology are emerging as mainstay to treat disease in the most effective way possible, allowing weaknesses in tumour biology to be exploited therapeutically. However, high drug attrition rates remain a challenge when developing new treatments in ALL. High drug attrition has been attributed to preclinical models not being clinically translatable. Key barriers hindering bench to bedside translation when using mouse models include species specificity, high financial costs, and lengthy experiments. Not all leukaemia samples engraft in mice. We have shown, in a previous study, that the 2D MSC co-culture system can successfully culture acute myeloid leukaemia (AML) cells from t(8;21)-positive samples, ^7^ which failed to engraft any mouse model available at the time. Moreover, samples that do engraft form successful xenograft models, yielding PDX samples in 3-6 months at which point drug testing can be started, which subsequently takes another 3-4 weeks. Indeed, although the time taken to generate a novel PDX-ALL sample can span between months to a year, this time is often shortened when cells from established PDX-ALL samples are serially transplanted in subsequent mouse models, to aid the generation of PDX-ALL lines. Nevertheless, some samples, particularly those belonging to good risk groups, such as high hyperdiploid samples, still require between 6 months – 1 year to engraft successfully before any drug response testing can begin. Moreover although *in vivo* toxicity assays are not needed for drugs being re-purposed, indicating this to be an area where animal-replacement technologies may perhaps not be relevant, animal testing has been required prior to regulatory approval being given for first-in-human studies. Furthermore, toxicity studies which use animal models are indicated when testing new combinatorial treatment regimens comprising repurposed drugs. This is because tolerability of drug combinations can be distinct from that of component single drugs.

Nevertheless, the U.S. Food and Drug Administration (FDA) has recently announced that new drugs would not be mandated to be tested in animals prior to first-in-human trials, and an FDA-wide program is furthermore prioritising development of human-relevant methods, with high *in vivo* predictivity, to replace, reduce and refine animal models in drug testing. This further corroborates the timeliness of prioritising development of transformative human cell-based, non-animal technologies towards preclinical drug testing. Most specifically, a key advantage of our prototype approach would be in medium-high throughput screening of single and combinatorial compounds, particularly as pre- *in vivo* screens, to prevent “false positive” 2D *in vitro* hits, such as that observed in Figure 4, from progressing towards *in vivo* drug efficacy testing.

A 3D BM-mesenchyme-leukaemia spheroid model will provide researchers with a tractable platform with improved biomimicry, where clinical samples can be cultured within the context of their mesenchymal microenvironment, and patient specific drug testing can be performed within shorter time periods, such as a week. In circumstances where sourcing primary MSC may be a challenge, human induced pluripotent stem cell (iPSC) differentiation into MSC may be an attractive alternative. ^12^ Besides delivering drug response data within clinically relevant timeframes the relative simplicity of human-cell-based spheroid models mean that they are tractable, transferrable, and sustainable. Consequently, such models have wide applications in translational research in academia and industry alike and can be set up in laboratories and SMEs locally, nationally and globally, especially in organisations that do not contain infrastructure for animal procedures. Following further extensive validation, such 3D-based preclinical models would furthermore have the potential to be embedded within clinical trials and healthcare systems for the purposes of detecting responders as well as to aid risk stratification. In this paper we show that our 2D model itself brought about significant animal replacement locally. Leukaemia research at Newcastle requires approximately 3600 animal procedures every year. Local metrics from previous studies confirmed that using the 2D pilot approach we replaced mice in approximately 67 drug tests each year. Given minimum of 40 animals are needed per drug test we replaced 2680 animals (out of 3600) which constituted a minimum 73% local animal replacement. To improve biomimicry and consequently translatability and transferability of our model, here we develop 3D BM-mesenchyme spheroids. We show that these 3D spheroids show superior ability in supporting culture of patient-derived ALL samples. We also show that sensitivity of leukaemia cells to drugs such as dexamethasone is reduced when these cells are being co-cultured with 3D spheroids. This means that compared to 2D MSC cultures, MSC spheroid-based models, might be promising to study the biology of leukaemia cells from relapsed disease, consequently helping interrogate treatment resistance mechanisms. Treatment resistance remains a major clinical challenge in cancer management. This simple and tractable 3D prototype will form the first steppingstone in developing next generation, scalable 3D preclinical models with improved *in vivo* and patient drug response predictivity. Such sustainable *ex vivo* models will ultimately aim to successfully replace existing moderate severity animal procedures in leukaemia research with clinically relevant human cell-based models that are able to successfully impact positive clinical outcome.

### Study limitations

Here we use primary human MSC, which indeed may be difficult to source, and which may lead to donor variability. Using iPSC derived MSC to co-culture patient-derived leukaemia ^12^ may be a more suitable alternative, and would furthermore enable assay standardisation, thereby improving data reproducibility. Moreover, although we show that MSC showed no adverse effect following dexamethasone and ABT-199 treatment, many anti-cancer treatments notably cytotoxic chemotherapy may cause direct toxicity to the MSC. However, this further emphasises the need to include key microenvironment cell types when conducting anti-cancer drug testing, especially given cancer treatments influence interactions between leukaemia and their surrounding niche, with many cytotoxic treatments affecting both leukaemia and BM cells in patients. Furthermore, we acknowledge that an ideal representation of 3D biomimetic human cell-based model would include capturing both mesenchymal and vascular BM components within an immune-responsive context.

## Data availability

Mendeley Data. A human mesenchymal spheroid prototype to replace moderate severity animal procedures in leukaemia drug testing. https://doi.org/10.17632/56npmkbpfb.1. ^24^

This project contains the following underlying data:

• **-** 3D spheres.tif
• **-** 3D Sphere.tif
• **-** drug combination.xlsx
• **-** *in vivo* Vs *in vitro* costs.xlsx
• **-** Raw Figure Data.xlsx

Data are available under the terms of the Creative Commons Attribution 4.0 International license (CC-BY 4.0).
