# Supplementary material for: A human mesenchymal spheroid prototype to replace moderate severity animal procedures in leukaemia drug testing
Source: F1000Res. 2023 Nov 6;11:1280. Originally published 2022 Nov 9. [Version 2] doi: 10.12688/f1000research.123084.2 (PMC10691310; doi:10.12688/f1000research.123084.2)
Supplement: Peer review history

# Reviewer report: Reviewer response for version 2

**DOI:** 10.5256/f1000research.157299.r221080

**Published:** 2023-11-23

**Authors:** Helen Wheadon

**Affiliations:** 1 Paul O'Gorman Leukaemia Research Centre, School of Cancer Sciences, University of Glasgow, Glasgow, UK

The authors have addressed all the comments and included the additional data requested.

Is the work clearly and accurately presented and does it cite the current literature?

Yes

If applicable, is the statistical analysis and its interpretation appropriate?

Not applicable

Are all the source data underlying the results available to ensure full reproducibility?

Yes

Is the study design appropriate and is the work technically sound?

Yes

Are the conclusions drawn adequately supported by the results?

Partly

Are sufficient details of methods and analysis provided to allow replication by others?

Partly

Reviewer Expertise:

Stem cells, leukaemia modelling, signalling, pre-clinical drug testing.

I confirm that I have read this submission and believe that I have an appropriate level of expertise to confirm that it is of an acceptable scientific standard.

# Reviewer report: Reviewer response for version 2

**DOI:** 10.5256/f1000research.157299.r221081

**Published:** 2023-11-20

**Authors:** Simon E. Richardson

**Affiliations:** 1 Wellcome - MRC Cambridge Stem Cell Institute, Cambridge, UK

Many thanks for accommodating my recommendations.

Is the work clearly and accurately presented and does it cite the current literature?

Yes

If applicable, is the statistical analysis and its interpretation appropriate?

Not applicable

Are all the source data underlying the results available to ensure full reproducibility?

Yes

Is the study design appropriate and is the work technically sound?

Partly

Are the conclusions drawn adequately supported by the results?

Partly

Are sufficient details of methods and analysis provided to allow replication by others?

Yes

Reviewer Expertise:

Leukaemia biology, disease modelling, stem cells, epigenetics, preclinical drug testing.

I confirm that I have read this submission and believe that I have an appropriate level of expertise to confirm that it is of an acceptable scientific standard.

# Reviewer report: Reviewer response for version 1

**DOI:** 10.5256/f1000research.135152.r159730

**Published:** 2023-01-31

**Authors:** Helen Wheadon

**Affiliations:** 1 Paul O'Gorman Leukaemia Research Centre, School of Cancer Sciences, University of Glasgow, Glasgow, UK

The manuscript by Wilson *et al.* provides a convincing argument for the requirement of additional in vitro models to improve drug testing in acute lymphocytic leukaemia (ALL) and reduce the reliance on Xenograft mouse models. Preliminary data is presented supporting the use of 3D human mesenchymal stroma cell (MSC) spheroid/primary ALL co-cultures for the use in preclinical testing of drugs. Proof of principle data is provided showing that simple 2D co-culture on MSCs gives a similar range of drug responses to dexamethasone and the BCL2 mimetic drug ABT199 as observed in in vivo studies for 2 primary ALL patient samples. The authors then show optimisation data using Aggrewell plates, demonstrating uniform spheroid formation and a corresponding increase in spheroid diameter when cell seeding density is increased. The final figure then compares primary ALL cell proliferation in 2D and 3D culture and drug responses to dexamethasone. Using 3 independent patient samples they show that MSC spheroids support cell proliferation over a 5 day time frame and provide protection to dexamethasone. These are important observations and support the introduction of a more physiologically relevant humanised system for drug screening prior to conducting in vivo studies. Another potential advantage would be if the authors had data demonstrating that the system supports patients' samples which do not engraft, as there is a high engraftment failure rate in Xenograft models with not all patient samples being successful. This could potentially lead to more robust data on patient’s responses. Given patient heterogeneity mouse models may be biasing certain traits/genetics due to the engraftment limitations observed.

 Overall, the system if adopted by other researchers could potentially reduce animal numbers and suffering, reduce the cost and select the most promising drugs for additional testing. It would therefore have impact on replacement and reduction of mouse experiments.

 However, there are some limitations in the results shown that would be strengthened by additional experiments:

1. The MSC are characterised by gene expression, it would be good to include flow cytometry data; CD90 ^+^105 ^+^73 ^+^34 ^-^45 ^-^ are key markers demonstrating stemness. The culture conditions using FCS can also lead to changes in the MSCs so it would be good to include passage number. Figure 3 live/dead staining of the spheroids especially for the size used in the drug treatment assays in Figure 4 would be important to include.
2. Data showing that dexamethasone and ABT199 do not detrimentally affect the MSC monoculture and MSC spheroids would also be important to include.
3. Figure 4 looks at proliferation, apoptosis assays would be good to include to show whether cell death was occurring.
4. Data showing similar results in ALL patient samples known to fail to engraft in vivo would strengthen the rationale for adopting the 3D system.
5. Although the potential costs and animal savings presented are feasible, the system is quite simplistic and is more likely to be incorporated as an additional test rather than be adopted as a replacement. It would be more attractive if the time frame could be extended and more details on the application for high throughput screening development was discussed. Also, in vivo toxicity testing of novel compounds will still be required in order for regulatory approval for any Phase 1 clinical trial in humans to be progressed.

Minor corrections:

**Research highlights **- blasts would only be appropriate for certain types of leukaemia, either need to define it’s ALL specifically or change to stem/progenitor.

**Scientific benefit:**

 A 3D spheroid-based approach with improved clinical relevance for ex vivo co-culture of primary patient leukaemia samples for further investigation into cancer biology such as stem/progenitor cell-niche interactions, stem/progenitor cell proliferation and treatment resistance.

**3Rs benefit:**

 To replace moderate severity animal (mice) procedures in leukaemia research and early pre-clinical drug testing.

**Current applications:**

 Exploration of leukaemia biology such as stem/progenitor cell proliferation, stem/progenitor cell -niche interactions, niche-impacted treatment resistance and obtain drug response data.

**Introduction:**

- Need to define at the start that you are talking about ALL as it reads like a general overview which means some of the % incidence rates etc. are not correct. It would be good to mention what type of ALL as well, is it B-ALL, T-ALL, childhood, adult?
- Paragraph 3 page 3: Most research groups would use primary patient samples in vitro prior to moving to in vivo models. This should be included.
- Paragraph 2 Page 4: Remove blasts and replace with LSCs also include disease type chronic myeloid leukaemia.
- Page 11 ‘such spheroid biobanks’: do they actually exist? Or are you planning on constructing a biobank?
- ‘MSC spheroids provide superior protection to leukaemia cells against dexamethasone treatment. are appropriate in modelling treatment resistance which remains a major clinical challenge in cancer management’.
  Is this statement correct, not really looking at resistance, just reduced sensitivity to the drugs at that dose when in 3D rather than 2D culture. Need to amend and extend to include how you would look at resistance i.e. relapsed patients and the potential to interrogate resistance mechanisms.

Is the work clearly and accurately presented and does it cite the current literature?

Yes

If applicable, is the statistical analysis and its interpretation appropriate?

Not applicable

Are all the source data underlying the results available to ensure full reproducibility?

Yes

Is the study design appropriate and is the work technically sound?

Yes

Are the conclusions drawn adequately supported by the results?

Partly

Are sufficient details of methods and analysis provided to allow replication by others?

Partly

Reviewer Expertise:

Stem cells, leukaemia modelling, signalling, pre-clinical drug testing.

I confirm that I have read this submission and believe that I have an appropriate level of expertise to confirm that it is of an acceptable scientific standard, however I have significant reservations, as outlined above.

# Reviewer report: Reviewer response for version 1

**DOI:** 10.5256/f1000research.135152.r158193

**Published:** 2022-12-19

**Authors:** Simon E. Richardson

**Affiliations:** 1 Wellcome - MRC Cambridge Stem Cell Institute, Cambridge, UK

This manuscript by Wilson et al provides a rationale, methodological details and pilot data supporting the use of 3D human mesenchymal stroma cell (MSC) spheroid co-cultures in the preclinical testing of drugs for acute leukaemia. The authors provide a detailed rationale for the potential benefits of this system, including: i) reduction in animal use/suffering; ii) reduced costs; and iii) the ability to model the protective impact of bone marrow micro-environmental influences in abrogating drug efficacy in vitro, reducing the number “false positive” drug hits taken forward to in vivo model systems. The authors provide data showing the equivalence of 2D in vitro drug sensitivity with PDX models (n=2) and compare 2D vs 3D co-culture of human leukaemias (n=3) in proliferation and dexamethasone sensitivity assays, showing consistently enhanced proliferation and reduced dexamethasone sensitivity in the 3D system. They also present methodological data on the establishment of MSC spheroids as well as modelling on the potential reduction in animal use and costs based on local experience.

 Overall, the rationale to develop more physiologically relevant in vitro co-culture systems is a very worthwhile endeavour with clear potential benefits for animal welfare, costs and in streamlining drug development. It also has the potential advantage of being used in the study of leukaemias that do not engraft in current immunosuppressed mouse models.

 In my opinion the work has three major limitations that would benefit from either additional data or acknowledgement in the discussion.

  

1. Whilst this work demonstrates the feasibility of the approach, it is not possible from the data presented to conclude that 3D systems perform better than either 2D co-culture or PDX systems. Specifically, the data presented uses a small number of patient samples (n=2 or 3) and drugs (Dexamethasone ± ABT199). The three systems are not directly compared and no example of an in vitro drug hit that proves “false-positive” when tested in vivo is tested in parallel 2D/3D co-culture.
2. There are a number of potential technical limitations of the co-culture approach that are not acknowledged: i) the requirement for access to a source of MSCs which are not readily available to most labs; ii) certain drugs (notably cytotoxic chemotherapy) are likely to have direct toxicity to the MSCs themselves; iii) the lack of a vascular component to this system might result in artificially high levels of physical protection from drug exposure by the 3D niche (i.e. potential for false negatives).
3. The potential costs and animal savings presented are feasible, but likely at the upper end of the spectrum and will differ between institutions. For example, the time to generate a novel PDX is indeed many months, but once established PDX models in ALL engraft much more quickly and reproducibly and established PDX material is readily available. In vivo toxicity testing is not needed for established drugs being re-purposed; conversely novel chemical agents will still need some in vivo toxicity data prior to regulatory approval for first-in-human studies.

Minor points:

1. In the introduction it would be helpful to specify the precise leukaemia being studied and at which age. In particular, relapse rates in adult B-ALL are much higher than those presented, which are indicative of the paediatric setting.
2. In the 2D co-culture methods, please provide a reference on how to process MSCs and specify the final volume of media per well used to plate the leukaemia cells.
3. For the response data presented in figures 1 and 4, please clarify in the legend or methods the nature of the readouts and timepoints used.

Is the work clearly and accurately presented and does it cite the current literature?

Yes

If applicable, is the statistical analysis and its interpretation appropriate?

Not applicable

Are all the source data underlying the results available to ensure full reproducibility?

Yes

Is the study design appropriate and is the work technically sound?

Partly

Are the conclusions drawn adequately supported by the results?

Partly

Are sufficient details of methods and analysis provided to allow replication by others?

Yes

Reviewer Expertise:

Leukaemia biology, disease modelling, stem cells, epigenetics, preclinical drug testing.

I confirm that I have read this submission and believe that I have an appropriate level of expertise to confirm that it is of an acceptable scientific standard, however I have significant reservations, as outlined above.
